# Systematic review of the relation between smokeless tobacco and cancer in Europe and North America

**DOI:** 10.1186/1741-7015-7-36

**Published:** 2009-07-29

**Authors:** Peter N Lee, Jan Hamling

**Affiliations:** 1 PN Lee Statistics and Computing Ltd, Surrey, UK

## Abstract

**Background:**

Interest is rising in smokeless tobacco as a safer alternative to smoking, but published reviews on smokeless tobacco and cancer are limited. We review North American and European studies and compare effects of smokeless tobacco and smoking.

**Methods:**

We obtained papers from MEDLINE searches, published reviews and secondary references describing epidemiological cohort and case-control studies relating any form of cancer to smokeless tobacco use. For each study, details were abstracted on design, smokeless tobacco exposure, cancers studied, analysis methods and adjustment for smoking and other factors. For each cancer, relative risks or odds ratios with 95% confidence intervals were tabulated. Overall, and also for USA and Scandinavia separately, meta-analyses were conducted using all available estimates, smoking-adjusted estimates, or estimates for never smokers. For seven cancers, smoking-attributable deaths in US men in 2005 were compared with deaths attributable to introducing smokeless tobacco into a population of never-smoking men.

**Results:**

Eighty-nine studies were identified; 62 US and 18 Scandinavian. Forty-six (52%) controlled for smoking. Random-effects meta-analysis estimates for most sites showed little association. Smoking-adjusted estimates were only significant for oropharyngeal cancer (1.36, CI 1.04–1.77, *n *= 19) and prostate cancer (1.29, 1.07–1.55, *n *= 4). The oropharyngeal association disappeared for estimates published since 1990 (1.00, 0.83–1.20, *n *= 14), for Scandinavia (0.97, 0.68–1.37, *n *= 7), and for alcohol-adjusted estimates (1.07, 0.84–1.37, *n *= 10). Any effect of current US products or Scandinavian snuff seems very limited. The prostate cancer data are inadequate for a clear conclusion.

Some meta-analyses suggest a possible effect for oesophagus, pancreas, larynx and kidney cancer, but other cancers show no effect of smokeless tobacco. Any possible effects are not evident in Scandinavia. Of 142,205 smoking-related male US cancer deaths in 2005, 104,737 are smoking-attributable. Smokeless tobacco-attributable deaths would be 1,102 (1.1%) if as many used smokeless tobacco as had smoked, and 2,081 (2.0%) if everyone used smokeless tobacco.

**Conclusion:**

An increased risk of oropharyngeal cancer is evident most clearly for past smokeless tobacco use in the USA, but not for Scandinavian snuff. Effects of smokeless tobacco use on other cancers are not clearly demonstrated. Risk from modern products is much less than for smoking.

## Background

Over the last 10 years, interest in smokeless tobacco (ST) as a possible safer alternative to smoking has risen. Although a number of recent reviews have considered the evidence relating ST to cancer, some have not included meta-analyses [1-3], and others have only provided quantitative summaries for specific sites: oropharyngeal cancer [4], pancreatic cancer [5], or oropharyngeal, oesophageal, pancreatic and lung cancer [6]. No formal comparisons have been conducted with the well-known effects of smoking [7,8].

The review described in this paper is restricted to studies in Western populations. In practice this predominantly means studies in the USA and Sweden, the only North American and European countries where the two major types of ST – chewing tobacco and snuff – are commonly used [2]. Although ST is also widely used in developing countries, particularly parts of Central and South-East Asia, the tobacco is often used in combination with other products, such as betel nut quid, slaked lime, areca nut and even snail shells [1,2,9]. This review also does not consider the limited data on nicotine chewing gum.

Our first objective is to carry out a comprehensive review of the available epidemiological evidence in Western countries relating ST to cancer, including meta-analyses for as many cancer types as the data justify. In meeting this objective, we take proper account of the potential confounding role of smoking by distinguishing effect estimates which are unadjusted for smoking and those which take smoking into account (either by adjustment in analyses based on the whole population of smokers and non-smokers combined or by restricting analysis to lifelong never smokers). Our second objective is to provide a quantitative indication of the relative effects of ST and cigarette smoking.

## Methods

### Study identification and selection

All reports had to satisfy the following inclusion criteria: published in a peer reviewed journal or the results publicly available, epidemiological study in humans, of cohort or case-control design, study location specified, any form of cancer as the outcome, and chewing tobacco, oral snuff or unspecified ST as the exposure. They also had to fall outside the exclusion criteria: conducted in an Asian or African population, no control group, or inappropriate design (case report, qualitative study or review/meta-analysis). Relevant papers were sought from a MEDLINE search conducted in May 2008 of "cancer" AND ("smokeless tobacco" OR "chewing tobacco" OR "snuff" OR "snus"), supplemented by citations in recent reviews [1-6,10] and in the papers obtained.

### Data extraction

Reports were grouped by study, and for each study details were abstracted (see Tables 1 and 2[11-114]) relating to the design, period, location, controls used and size, the exposure (method of assessment, type of ST, exposure doses and durations considered), the outcome (cancer sites studied) and issues relating to analysis (type of effect measure, analysis methods, extent of adjustment for smoking and other factors, and availability of dose-response data). The extent of adjustment for smoking for a study was categorised into five groups: A. *no information *– effect estimates are provided but no details are given of any adjustments made; B. *no adjustment *– effect estimates are available for the whole population, but smoking is not taken into account; C. *never smokers *– the only effect estimates available are for never smokers; D. *some adjustment *– effect estimates adjusted for smoking are available, but the adjustment is relatively simple, using two or three level broad groupings (for example, ever/never smoked, current/non-current smoker, current/former/never smoker), and takes no account of daily amount smoked or duration of smoking; and E. *more adjustment *– effect estimates are available that take into account daily amount smoked, duration of smoking and/or their product (pack-years). Studies were categorised under D or E if smoking-adjusted effect estimates are available, regardless of whether some results for never smokers are also presented. The method used to adjust for smoking is not always clear. Studies where the authors merely report that they 'adjusted for cigarette smoking' are included in category D.

**Table 1:** Cohort studies of smokeless tobacco and cancer

Study	Country	Follow-up period	Baseline population	Exposure^a^	Reference^b^	Cancers studied (cases)^c^
Lutheran Brotherhood cohort^d^	USA	1966 to 1986	17,633 white men aged 35+ years	ST	Hsing *et al*. 1990 [11]	Prostate (149)
					Kneller *et al*. 1991 [12]	Stomach (75)
					Zheng *et al*. 1993 [13]	Pancreas (57)
US Veterans cohort^e^	USA	1954/57 to 1980	248,046 US veterans aged 31–84 years, over 99.5% men	ST	Hsing *et al*. 1991 [15]	Prostate (4,607)
					Heineman *et al*. 1992 [16]	Multiple myeloma (582)^f^
					Zahm *et al*. 1992 [17]	Soft tissue sarcoma (119), pharynx (55), buccal cavity (74)
					Heineman *et al*. 1995 [18]	Colon (3,812), rectum (1,100)
Iowa cohort	USA	1986/89 to 1995	1,572 men aged 40+ years, controls in a case-control study	ST	Putnam *et al*. 2000 [20]	Prostate (101)^f^
NHANES I follow-up cohort^g^	USA	1971/75 to 2002	14,407 adults aged 25–74 years^h^	ST	Accort *et al*. 2002 [21]	All, lung^i^
					Accort *et al*. 2005 [22]	All, lung, breast, digestive, oral, prostate^f, i^
CPS-I^j^	USA	1959 to 1972	77,407 never smoking men aged 30+ years from 25 states	ST	Henley *et al*. 2005 [23]	All (2,332), oral (13), digestive (913), lung (134), genitourinary (559)
CPS-II^k^	USA	1982 to 2000	114,809 never smoking men aged 30+ years nationwide	ST^l^	Henley *et al*. 2005 [23]	All (6,140), oral (46), digestive (1,999), lung (400), genitourinary (1,709), haematopoietic (923)
		1982 to 1996	467,788 men aged 30+ years nationwide	ST	Chao *et al*. 2002 [24]	Stomach (996)
Norway cohorts^m^	Norway	1966 to 2001	10,136 men from two cohorts, a sample of the 1960 census and relatives of Norwegian migrants to the USA	Snuff	Boffetta *et al*. 2005 [26]	Oral (34), oesophagus (27), stomach (217), pancreas (105), lung (343), kidney (88), bladder (239)^n^
Swedish construction workers	Sweden	1974 to 1985	135,036 men	Snuff	Bolinder *et al*. 1994 [28]	All (1,269), lung (204)
		1971 to 2000	337,311 men		Odenbro *et al*. 2005 [29]	Cutaneous squamous cell carcinoma (756)^f^
		1971 to 2000	335,612 adults, over 99.3% men		Fernberg *et al*. 2006 [30]	Malignant lymphoma (1,514)^f^
		1971 to 2004	336,381 men		Fernberg *et al*. 2007 [31]	Leukaemia (372), multiple myeloma (520)^f^
		1978 to 2004	279,897 men		Luo *et al*. 2007 [32]	Oral (248), lung (2,198), pancreas (448)^f^
		1971 to 2004	339,802 men		Odenbro *et al*. 2007 [33]	Melanoma (1,639)°
		1971 to 2004	336,381 men		Zendehdel *et al*. 2008 [34]	Stomach (1,385), oesophagus (366)^f^
Uppsala County cohort	Sweden	1973/74 to 2002	9,976 men	Snuff	Roosaar *et al*. 2008 [35]	All (1,572), smoking-related (493), oral (34)^p^

^a ^Only exposures for which results are available are shown.

^b ^Main references. Other references supplying limited data are indicated in footnotes.

^c ^Numbers of cases are totals for the sexes specified. Numbers of cases exposed to ST are shown in the tables presenting results by site. Cases are deaths, unless indicated. Oral is used as an abbreviation for oropharnyx.

^d ^Some limited additional results for the Lutheran Brotherhood cohort, based on follow-up to 1981, were reported earlier for cancers of the prostate, pancreas and oesophagus in IARC Monograph 37 in 1985 [14].

^e ^Some limited additional results for the US Veterans cohort, based on follow-up from 1954 to 1969 were presented earlier for a range of cancers in an abstract by Winn *et al*. in 1982 [19].

^f ^Cancers listed are incident cases.

^g ^NHANES I = First National Health and Nutrition Examination Survey.

^h ^Data on ST use were only collected in 3,847 subjects at baseline in 1971–1975, but were collected for all subjects in follow-up surveys in 1982–1984. 6,805 subjects were considered in the mortality analyses [21] and 7,779 in the incidence analyses [22].

^i ^Numbers of cases not given.

^j ^CPS-I = Cancer Prevention Study I.

^k ^CPS-II = Cancer Prevention Study II. Some additional results for lung cancer, based on mortality to 2002, comparing 111,952 men who quit cigarette smoking with 4,443 who switched to ST, were presented by Henley *et al*. in 2007 [25].

^l ^Results for chewing and snuff are also given for all cancers and lung cancers.

^m ^Some limited additional results, based on follow-up to 1978, were reported by Heuch *et al*. in 1983 [27] for pancreatic cancer incidence and in IARC Monograph 37 in 1985 [14] for cancers of the buccal cavity/pharynx, oesophagus, pancreas and prostate.

^n ^Cancers listed include incident cases.

° Includes cutaneous malignant melanoma, melanoma *in situ *and intraocular malignant melanoma.

^p ^Numbers are incident cases. An analysis of overall cancer based on 1,574 deaths was also conducted.

ST = smokeless tobacco.

**Table 2:** Case-control studies of smokeless tobacco and cancer

Study	Country	Study period^a^	Controls^a^	Sex^b^	Exposures studied^c^	Cancers studied (cases)^d^
Broders 1920 [37]	USA	NA	Hospital	M+F	Chew, snuff, ST	Oral (537)
Doll and Hill 1952 [38]	UK	1948–1952	Hospital	M	Chew, snuff	Lung (1,209)
Moore *et al*. 1953 [39]	USA	1951–1952	Hospital	M	ST	Oral (112), face (93)
Wynder *et al*. 1957 [40]	Sweden	1952–1955	Hospital	M	Chew	Oral (166), oesophagus (39), larynx (60)
Wynder and Bross 1957 [41]	USA	NA	Hospital	M	Chew	Oral (543)
Peacock *et al*. 1960 [42]	USA	1952–1958	Hospital	M+F	ST	Oral (45)
Lockwood 1961 [43]	Denmark	1942–1956	Population	M+F	ST	Bladder (282)
Wynder and Bross 1961 [44]	USA	1956–1959	Hospital	M	Chew	Oesophagus (150)
Vogler *et al*. 1962 [36]	USA	1956–1957	Hospital	M+F	Chew, snuff	Oral (228)
Vincent and Marchetta 1963 [45]	USA	NA	Hospital	M	Snuff	Oral (66), larynx (23)
Wynder *et al*. 1963 [46]	USA	1957–1960	Hospital	M	Chew, snuff, ST	Bladder (300)
Bennington and Laubscher 1968 [47]	USA	1951–1956	Hospital	M	Chew	Kidney (88)
Dunham *et al*. 1968 [48]	USA	1958–1964	Hospital	M+F	ST	Bladder (493)
Martinez 1969 [49]	Puerto Rico	1966	Hospital, population	M+F	Chew	Oral (221), oesophagus (179)
Keller 1970 [50]	USA	1958–1962	Hospital	M	ST	Oral (314)
Cole *et al*. 1971 [51]	USA	1967–1968	Population	M+F	Chew, snuff	Bladder (470)
Bjelke *et al*. 1974 [52]	USA	NA	NA	NA	Chew	Colorectal (373), oesophagus (52), stomach (83)
	Norway	NA	NA	NA	Chew	Colorectal (278), stomach (228)
Armstrong *et al*. 1976 [53]	UK	1972–1974	Hospital	M	ST	Kidney (96)
Browne *et al*. 1977 [54]	UK	1957–1971	Population	M+F	Chew	Oral (75)
Williams and Horm 1977 [55]	USA	1969–1971	Hospital	M+F	ST	Many types (7,518)^e^
Wynder and Stellman 1977 [56]	USA	1969–1975	Hospital	M	Chew, snuff, ST	Oral (593), bladder (589), larynx (387), lung (1,051), oesophagus (183)
Engzell *et al*. 1978 [57]	Sweden	1961–1971	Population	M	Snuff	Nose (36)
Howe *et al*. 1980 [58]	Canada	1974–1976	Population	M	Chew	Bladder (480)
Westbrook *et al*. 1980 [59]	USA	1955–1975	Hospital	F	Snuff	Oral (55)
Pottern *et al*. 1981 [60]	USA	1975–1977	Decedent	M	Chew, snuff	Oesophagus (120)
Winn *et al*. 1981 [61]	USA	1975–1978	Hospital	F	Snuff	Oral (255)
Mommsen and Aagaard 1983 [62]	Denmark	1977–1980	Population	M	Chew	Bladder (165)
Wynder *et al*. 1983 [63]	USA	1977–1980	Hospital	M	Chew, snuff, ST	Oral (414)
Brinton *et al*. 1984 [64]	USA	1970–1980	Hospital, decedent	M+F	Chew, snuff, ST	Nose (160)
McLaughlin *et al*. 1984 [65]	USA	1974–1979	Population	M	Chew, snuff, ST	Kidney (313)
Hartge *et al*. 1985 [66]	USA	1977–1978	Population	M	Chew, snuff, ST	Bladder (2,240)
Weinberg *et al*. 1985 [67]	USA	1978–1980	Decedent, population	M	Chew	Stomach (178)
Goodman *et al*. 1986 [68]	USA	1977–1983	Hospital	M+F	Chew	Kidney (267)
Kabat *et al*. 1986 [69]	USA	1976–1983	Hospital	F	Snuff	Bladder (152)
Stockwell and Lyman 1986 [70]	USA	1982	Population	M+F	ST	Oral (1,462), nose (92), larynx (161)
Young *et al*. 1986 [71]	USA	4 yr period	Hospital	M+F	ST	Oral (317), larynx (179)
Lindquist *et al*. 1987 [72]	Sweden	1980–1983	Population	M	Snuff	Leukaemia (76)
Asal *et al*. 1988 [73]	USA	1981–1984	Hospital, population	M	Snuff	Kidney (209)
Blot *et al*. 1988 [74]	USA	1984–1985	Population	M+F	ST	Oral (1,114)
Falk *et al*. 1988 [75]	USA	1979–1983	Hospital	M+F	Chew, snuff	Pancreas (363)
Morris Brown *et al*. 1988 [76]	USA	1982–1984	Population	M	ST	Oesophagus (207)
Slattery *et al*. 1988 [77]	USA	1977–1983	Population	M	Chew, snuff, ST	Bladder (332)
Spitz *et al*. 1988 [78]	USA	1985–1987	Hospital	M+F	Chew, snuff, ST	Oral (185)^f^
Burch *et al*. 1989 [79]	Canada	1979–1982	Population	M	Chew, snuff	Bladder (627)
Franco *et al*. 1989 [80]	Brazil	1986–1988	Hospital	M+F	ST	Oral (232)
Zahm *et al*. 1989 [81]	USA	1976–1982	Population	M	ST	Soft tissue sarcoma (133)
Farrow *et al*. 1990 [82]	USA	1982–1986	Population	M	Chew	Pancreas (148)
Blomqvist *et al*. 1991 [83]	Sweden	NA	Hospital	M+F	Snuff	Oral (61)
Ghadirian *et al*. 1991 [84]	Canada	1984–1988	Population	M+F	Chew	Pancreas (179)
Maden *et al*. 1992 [85]	USA	1985–1989	Population	M	ST	Oral (131)
Marshall *et al*. 1992 [86]	USA	1975–1983	Population	M+F	Chew	Oral (290)
Morris Brown *et al*. 1992 [87]	USA	1981–984	Population	M	ST	Leukaemia (578)
Morris Brown *et al*. 1992 [88]	USA	1981–1984	Population	M	ST	Non-Hodgkin's lymphoma (622)
Sterling *et al*. 1992 [89]	USA	1986	Population	M+F	Snuff, ST	All cancer (459,792), oral (6,976), all digestive (109,514)
Mashberg *et al*. 1993 [90]	USA	1972–1989	Hospital	M	Chew, snuff, ST	Oral (359)
Perry *et al*. 1993^g^	USA	About 1992	Hospital	M+F	ST	Oral (133)
Spitz *et al*. 1993 [92]	USA	1987–1991	Hospital	M+F	Chew	Oral (108)^f^
Chow *et al*. 1994 [93]	USA	1985–1997	Population	M	Chew	Bile duct (49)
Hansson *et al*. 1994 [94]	Sweden	1989–1992	Population	M+F	Chew, snuff	Stomach (338)
Hardell *et al*. 1994 [95]	Sweden	1974–1978	Population	M	Snuff	Non-Hodgkin's lymphoma (105)
Hayes *et al*. 1994 [96]	USA	1986–1989	Population	M	Chew, snuff, ST	Prostate (981)
Kabat *et al*. 1994 [97]	USA	1977–1990	Hospital	M+F	Chew, snuff	Oral (1,560)
Bundgaard *et al*. 1995 [98]	Denmark	1986–1990	Population	M+F	Chew	Oral (161)
McLaughlin *et al*. 1995 [99]	5 countries^h^	1989–1991	Population	M+F	ST	Kidney (1,732)
Muscat *et al*. 1995 [100]	USA	1977–1993	Hospital	M	Chew	Kidney (543)
Muscat *et al*. 1997 [101]	USA	1985–1993	Hospital	M	Chew, snuff	Pancreas (290)
Lewin *et al*. 1998 [102]	Sweden	1980–1989	Population	M	Snuff	Oral (266), larynx (157), oesophagus (122)
Muscat and Wynder 1998 [103]	USA	1977–1980	Hospital	M+F	Chew, ST	Oral (128)
Schildt *et al*. 1998 [104]	Sweden	1980–1989	Population	M+F	Chew, snuff, ST	Oral (410)
Schwartz *et al*. 1998 [105]	USA	1990–1995	Population	M	ST	Oral (165)
Yuan *et al*. 1998 [106]	USA	1986–1994	Population	M+F	ST	Kidney (1,204)
Ye *et al*. 1999 [107]	Sweden	1989–1995	Population	M+F	Chew, snuff	Stomach (514)
Lagergren *et al*. 2000 [108]	Sweden	1995–1997	Population	M+F	Snuff	Oesophagus (189), stomach (429)
Zheng *et al*. 2001 [109]	USA	NA	Population	M+F	Chew, snuff	Brain (375)
Schroeder *et al*. 2002 [110]	USA	1980–1982	Population	M	Chew, snuff, ST	Non-Hodgkin's lymphoma (182)
Alguacil and Silverman 2004 [111]	USA	1986–1989	Population	M+F	ST	Pancreas (526)
Bracci and Holly 2005 [112]	USA	1988–1993	Population	M	ST	Non-Hodgkin's lymphoma (725)
Rosenquist *et al*. 2005 [113]	Sweden	2000–2004	Population	M+F	Snuff	Oral (132)
Hassan *et al*. 2007 [114]	USA	2000–2006	Hospital	M+F	Chew, snuff, ST	Pancreas (808)

^a ^NA = not available.

^b ^M = male, F = female, M+F = both sexes. Studies of both sexes with results reported only for males are shown as M.

^c ^Only exposures for which results are available are shown.

^d ^Oral (oropharyngeal) is defined as in Weitkunat *et al*. 2007 [4] to include any of the following sites: buccal mucosa, floor of mouth, gingival, gum/palate, lip, oral cavity/mouth, pharynx/alveolus, tongue, tonsils, salivary glands and oral unspecified. This reference also shows the actual sites included for most of the studies included here. For other cancers, more precise definitions of site or histology are given, where relevant, in the tables presenting the findings. Numbers of cases are totals for the sexes specified. Numbers of cases exposed to ST are shown in the tables presenting results by site.

^e ^Results were presented for the following 'known tobacco-related' sites: oral (298 cases), oesophagus (72), larynx (119), lung (931) and bladder (306), with comparisons made with all other 'non-related' sites. Results were also presented for various non-related sites: stomach (266), small intestine (19), colon (722), rectum (339), liver (45), gall bladder/bile duct (81), pancreas (224) breast (1,177), cervix (266), uterus (38), ovary (180), vulva (31), prostate (531), male genitalia (53), kidney (126), connective tissue (84), melanoma (99), nervous system (136), thyroid gland (94), lymphosarcoma (121), Hodgkin's disease (84), other lymphomas (33), multiple myeloma (86), leukaemia (172) and other or unknown primaries (385), with comparisons made with all other non-related sites combined.

^f ^Includes larynx cancer.

^g ^"Attributable oral cancer risk due to smokeless tobacco use based on a case-control study at Sinai Hospital in Detroit"; Perry *et al*., unpublished. Cited by Gross *et al*. 1995 [91].

^h ^Australia, Denmark, Germany, Sweden and USA.

ST = smokeless tobacco.

Based on the availability of relevant data, 13 cancer groupings (oropharyngeal, oesophagus, stomach, pancreas, other digestive, larynx and nasal, lung, prostate, bladder, kidney, haematopoietic and lymphoid, other and all), were selected, with results for each grouping tabulated in a standard way, with details given of the source, exposure to ST, smoking group, sex, number of cases and adjustment factors for each effect estimate or indication of association (see tables dealing with individual effects estimates, below). For each study the intent is to extract the relative risk (RR) or odds ratio (OR) adjusted for the most factors, relevant to current, former or ever exposure to chewing tobacco, snuff or overall/undefined ST. Where relevant results for a study are reported in more than one paper, those based on the greatest number of cases are used.

Results are included, where available, for the whole population and for never smokers, and for sexes separately. RR or OR estimates based on zero exposed cases (or controls) are not included as providing too little information and because a valid confidence interval (CI) cannot be calculated. Suitable estimates of effect (RR or OR) and precision (CI) provided by the authors are used if possible, estimates otherwise being calculated from available data presented in the source publication, based on methods [115-118] summarised elsewhere [4]. Where an effect estimate cannot be calculated, statements made by the authors are summarised into terms such as 'no association' or 'no significant association'. Data are summarised for all types of cancer, except those relating to subdivision by type within site (for example, adenocarcinoma or squamous cell carcinoma of the lung, or t(14; 18)-positive and -negative non-Hodgkin's lymphoma or those relating to combined 'other' groups of cancers, which typically vary in definition from study to study).

### Data presentation

Study-specific results for the different types of cancer are presented in an essentially identical format, with a standard set of information included for each effect estimate included. Points to note about the entries in the various columns are discussed below.

#### Source

For the case-control studies, the source reference is shown. For the cohort studies, the source reference is also shown, but the study is also identified by name.

#### ST use – type

The exposure is identified as chewing tobacco ('chew'), 'snuff' or smokeless tobacco ('ST'). ST implies the results relate to smokeless tobacco unspecified by the author, or to use of either chewing tobacco or snuff or both.

#### ST use – exposure

Results are presented for current, former or ever use, or simply for 'use' where the timing of exposure was unspecified by the author. For current, former or ever use, the comparison is with never use; for use, it is with non-use.

#### Smoking

Results are presented only for any smoking (that is, based on the combined population of ever and never smokers) and for never smokers.

#### Sex

Results are shown, where available, for the sexes separately, though in some studies results are given only for the sexes combined.

#### RR/OR id

Within each table, each effect estimate (RR or OR) is given a unique identification number, so that those which are included in specific meta-analyses can readily be seen.

#### Cases

The number of ST-exposed cases is shown. Total numbers of cases are given elsewhere. Estimates are not presented unless there is at least one exposed case.

#### Estimate (95% CI)

This is the RR for cohort studies or the OR for case-control studies, together with its 95% CI. For many studies, the estimates are not given directly in the source paper, but were calculated from data provided. This involved one or more of the following: estimating numbers of exposed and unexposed cases and controls from proportions exposed given numerically or graphically and, where appropriate, combining numbers over level of exposure or cancer subtype; calculating estimates from a 2 × 2 table, or multiple independent 2 × 2 tables using standard methods [115], and calculating estimates from non-independent RR/ORs by level of exposure or by cancer type using the method of Hamling *et al*. 2007 [118]. Fuller details of the method of calculation used for each estimate are available on request. In a limited number of studies, as indicated in the tables, estimates were available separately for chewing tobacco and for snuff, but data were lacking for joint use. Here estimates for combined ST use were calculated assuming that no one used both chewing tobacco and snuff. Where there is a choice of relevant estimates from a study, preference is given to the estimate adjusted for the most potential confounding factors, and, for cohort studies, the estimate from the publication with a longer follow-up period.

#### Adjustment factors

The adjustment factors used for each estimate are shown. For matched case-control studies, the matching factors are not included unless the estimate specifically took this into account (for example, by conditional logistic regression). The factors used have been simplified into a relatively short consistent list, rather than repeating *verbatim *the wide variety of variable descriptions given by the original authors. Thus 'res' (area of residence) includes any variable based on the location of the subject and, for example, includes centre in multicentre case-control studies. 'Diet' includes any aspect of diet, and 'alc' (alcohol) any aspect of alcohol use. Estimates relevant to never smokers are not listed as being adjusted for smoking ('smok').

#### Layout

For the five columns, ST use – type, ST use – exposure, smoking, sex and adjustment factors, any blank entry for a particular effect estimate is assumed to be the same as in the first previous non-blank entry in that column. This avoids needless repetition and makes the tables easier to read.

### Meta-analysis

Estimates with no CI are not included in the meta-analyses. The standard error of the logarithm of estimates of effect size was calculated from its reported or estimated CI, assuming that the effect size was log-normally distributed. The logarithms of the effect sizes and their corresponding standard errors form the data points for fixed-effect and random-effects meta-analysis [116].

For most cancer groupings, results of nine random-effect meta-analyses are presented, subject to availability of data (see tables summarising meta-analysis results, below). In the first set of three, *any*, there is no restriction of estimates on type of exposure or region. In the second set, *any ST use (USA)*, estimates are restricted to those from studies conducted in the USA (or on occasion in Puerto Rico), while in the third set, *snuff (Scandinavia)*, estimates are restricted to those for snuff and for studies conducted in Scandinavia. Each of the three sets of meta-analyses is divided into *overall data*, *smoking-adjusted *and *never smokers*. In the *overall data *analyses, estimates are not restricted on smoking status or on adjustment for smoking. The *smoking-adjusted *analyses only include estimates that are for the whole population and adjusted for smoking or are for never smokers. The *never smokers *analyses are restricted to estimates for never smokers. For oropharyngeal cancer, where more estimates are available, some additional meta-analysis results are shown, based on estimates that are smoking and alcohol adjusted, and on estimates published since 1990.

To avoid double-counting multiple non-independent estimates from the same study, estimates from each study are selected for inclusion in the meta-analyses using order of preference lists for ST exposure (ever use/unspecified use/current use/former use), then smoking status (any – based on the combined population of smokers and non-smokers/never smokers), and then ST type (ST/snuff/chew), with each list being in order of most to least preferred. At each step we retain those estimates highest up the list, discarding any estimate lower in the preference order. If the procedure ends up with separate estimates for males and for females, both are included in the analysis. In one study [36], the results available are for males for chewing and for females for snuff (see Table 3). Although the procedure, strictly applied, selects only the snuff estimate, it was decided to include both in the relevant meta-analyses.

**Table 3:** Oropharyngeal cancer; individual effect (relative risk/odds ratio) estimates

	ST use				RR/OR			
				
Source^a^	Type^b^	Exposure^c^	Smoking	Sex	Id.	Cases^d^	Estimate (95%CI)	Adjustment factors^e^
**Cohort studies**								
								
US Veterans: Zahm *et al*. 1992 [17]	ST	Ever	Any	M^f^	1	129	4.11 (2.90–5.84)^g^	age, time
CPS-I: Henley *et al*. 2005 [23]	ST	Current	Never	M	2	4	2.02 (0.53–7.74)	age, alc, asp, bmi, diet, edu, exer, occ, race
CPS-II: Henley *et al*. 2005 [23]	ST	Current	Never	M	3	1	0.90 (0.12–6.71)	age, alc, asp, bmi, diet, edu, exer, occ, race
Norway Cohorts: Boffetta *et al*. 2005 [26]	Snuff	Current	Any	M	4	6	1.13 (0.45–2.83)	age, smok
		Former			5	3	1.04 (0.31–3.50)	
		Ever			6	9	1.10 (0.50–2.41)	
Swedish construction workers: Luo *et al*. 2007 [32]	Snuff	Ever	Any	M	7	NA	0.70 (0.50–0.90)	age, bmi, smok
		Current	Never		8	9	0.90 (0.40–1.80)	age, bmi
		Former			9	1	0.70 (0.10–5.00)	
		Ever			10	10	0.80 (0.40–1.70)	
Uppsala County: Roosaar *et al*. 2008 [35]	Snuff	Ever	Any	M	11	11	3.10 (1.50–6.60)	age, alc, res, smok, time
			Never		12	5	2.30 (0.70–8.30)	age, alc, res, time
								
**Case-control studies**								
Broders 1920 [37]	Chew	Use	Any	M+F	13	128	2.05 (1.48–2.83)^g^	smok
	Snuff				14	2	1.76 (0.12–26.52)^g^	none
	ST				15	130	2.05 (1.48–2.83)^g^	
Moore *et al*. 1953 [39]	ST	Use	Any	M	16	65	3.00 (1.37–6.54)^g^	none
Wynder *et al*. 1957 [40]	Chew	Ever	Any	M	17	NA	no association^h^	none
Wynder and Bross 1957 [41]	Chew	Ever	Any	M	18	91	2.00 (1.16–3.47)^g^	smok
Peacock *et al*. 1960 [42]	ST	Use	Any	M	19	14	3.06 (1.08–8.63)^g^	age, ins
				F	20	11	2.00 (0.66–6.01)^g^	
Vogler *et al*. 1962 [36]	Chew	Ever	Any	M	21	46	7.38 (4.31–12.62)^g^	none
	Snuff			F	22	54	38.28 (21.49–68.15)^g^	
Vincent and Marchetta 1963 [45]	Snuff	Use	Any	M	23	12	4.22 (1.41–12.63)^g^	none
Martinez *et al*. 1969 [49]	Chew	Use	Any	M	24	4	2.29 (0.62–8.48)^g^	none
				F	25	1	0.34 (0.04–2.79)^g^	
Keller 1970 [50]	ST	Use	Any	M	26	11	3.63 (1.02–12.95)^g^	smok
			Never		27	4	3.04 (0.62–14.99)^g^	
Browne *et al*. 1977 [54]	Chew	Use	Any	M+F	28	7	0.67 (0.27–1.66)^g^	none
Williams and Horm 1977 [55]	ST	Ever	Any	M	29	16	0.91 (0.53–1.56)^g^	none
				F	30	2	1.54 (0.37–6.42)^g^	
Wynder and Stellman 1977 [56]	Chew	Ever	Any	M	31	10	0.62 (0.32–1.21)^g^	none
	Snuff				32	61	1.15 (0.85–1.55)^g^	
	ST				33	71	1.02 (0.78–1.34)^i^	
Westbrook *et al*. 1980 [59]	Snuff	Ever	Any	F	34	50	540.00 (60.97–4782.82)^g^	none
Winn *et al*. 1981 [61]	Snuff	Ever	Any	F	35	107	2.67 (1.83–3.90)^g^	race, smok
Wynder *et al*. 1983 [63]	Chew	Ever	Any	M	36	37	1.00 (0.62–1.61)^g^	none
	Snuff				37	12	0.42 (0.11–1.65)^g^	
	ST				38	49	0.90 (0.57–1.41)^i^	
Stockwell and Lyman 1986 [70]	ST	Ever	Any	M+F	39	11	2.02 (1.01–4.02)^g^	none
Young *et al*. 1986 [71]	ST	Ever	Any	M	40	NA	no association	none
Blot *et al*. 1988 [74]	ST	Ever	Any	M	41	46	0.85 (0.57–1.26)^g^	none
				F	42	11	3.44 (1.09–10.91)^g^	
			Never	F	43	6	6.20 (1.90–19.80)	age, race, res, resp
Spitz *et al*. 1988 [78]	Chew	Ever	Any	M+F	44	23	1.00 (0.54–1.85)^g^	none
	Snuff				45	9	3.40 (1.00–10.90)	
	ST				46	25	1.05 (0.57–1.91)^g^	
Franco *et al*. 1989 [80]	ST	Use	Any	M+F	47	9	1.40 (0.59–3.33)^g^	none
Blomqvist *et al*. 1991 [83]	Snuff	Ever	Never	M+F	48	2	0.67 (0.08–5.75)^g^	none
Maden *et al*. 1992 [85]	ST	Ever	Any	M	49	19	4.50 (1.50–14.30)	age
Marshall *et al*. 1992 [86]	Chew	Use	Any	M	50	NA	no significant association	none
Sterling *et al*. 1992 [89]	ST	Ever	Any	M+F	51	28^g^	1.04 (0.41–2.68)^g^	age, alc, occ, race, sex, smok
	Snuff	Ever	Any	M+F	52	NA	2.42 (1.28–4.59)	age, race, sex
Mashberg *et al*. 1993 [90]	Chew	Ever	Any	M^f^	53	NA	1.00 (0.70–1.40)	age, alc, race, smok
	Snuff				54	NA	0.80 (0.40–1.90)	
	ST				55	52	0.96 (0.70–1.33)^i^	
Perry *et al*. 1993^j^	ST	Use	Any	M+F	56	10	1.43 (0.64–3.21)^g^	age, alc, occ, race, sex, smok
Spitz *et al*. 1993 [92]	Chew	Use	Any	M+F	57	NA	1.20 (not significant)	none
Kabat *et al*. 1994 [97]	Chew	Ever	Any	M	58	67	1.11 (0.81–1.53)^g^	smok
	Snuff	Ever	Never	M+F	59	4	4.79 (1.19–19.30)^g^	none
Bundgaard *et al*. 1995 [98]	Chew	Ever	Any	M+F	60	8	1.44 (0.59–3.51)^g^	none
Lewin *et al*. 1998 [102]	Snuff	Current	Any	M	61	18	0.84 (0.47–1.50)^g^	age, alc, res, smok
		Former			62	22	1.28 (0.70–2.35)^g^	
		Ever			63	40	0.98 (0.63–1.50)^g^	
Muscat *et al*. 1998 [103]	Chew	Ever	Any	M+F	64	3	0.89 (0.18–4.49)^g^	none
	ST				65	4	1.19 (0.26–5.45)^i^	
Schildt *et al*. 1998 [104]	Chew	Use	Any	M+F	66	5	0.60 (0.20–2.00)	age, sex, res
	Snuff	Current			67	39	0.70 (0.40–1.10)	
		Former			68	28	1.50 (0.80–2.90)	
		Ever			69	67	0.80 (0.50–1.30)	age, alc, sex, smok, res
		Current	Never		70	19	0.70 (0.40–1.20)	age, sex, res
		Former			71	9	1.80 (0.90–3.50)	
		Ever			72	28	1.01 (0.64–1.57)^g^	
	ST	Ever	Any		73	72	0.87 (0.61–1.25)^i^	none
Schwartz *et al*. 1998 [105]	ST	Ever	Any	M	74	NA	1.00 (0.40–2.30)	age, alc, smok
Rosenquist *et al*. 2005 [113]	Snuff	Current	Any	M+F	75	13	1.10 (0.50–2.50)	alc, smok
		Former			76	7	0.30 (0.10–0.90)	
		Ever			77	20	0.70 (0.30–1.30)	

^a ^Fuller details of the studies are given in Tables 1 and 2.

^b ^ST implies smokeless tobacco unspecified, or combined snuff use or chewing.

^c ^Ever, former and current ST use were compared with never ST. Use indicates timing not given and comparison is with non use.

^d ^'Id.' is the RR/OR identification number used in Table 4, and 'Cases' is the number of cases in ST users as defined. NA = not available.

^e ^Abbreviations used: alc = alcohol, asp = aspirin, bmi = body mass index, edu = education, exer = exercise, ins = insurance status, occ = occupation, res = area of residence, resp = respondent, smok = smoking.

^f ^The population included < 0.5% females.

^g ^RR/OR and/or 95% CI estimated from data provided in the source.

^h ^The average ridit duration of chewing did not differ significantly from the controls for any type of oral cancer.

^i ^RR/OR and/or 95% CI estimated from data provided in the source assuming that no one both chewed and used snuff.

^j ^"Attributable oral cancer risk due to smokeless tobacco use based on a case-control study at Sinai Hospital in Detroit", Perry *et al*., unpublished. Cited by Gross *et al*. 1995 [91].

CI = confidence interval; ST = smokeless tobacco; OR = odds ratio; RR = relative risk.

The presentation of the meta-analyses shows the number of estimates combined; the identification numbers of these estimates (so that they can be related to the preceding table of individual effect estimates); the combined random-effects estimate, with its 95% CI [116], the chi-squared and *P *value of homogeneity [119] and the I^2 ^statistic [120]. The meta-analyses conducted also include a test for publication bias [121] where five or more estimates are combined. Findings significant at *P *< 0.1 are indicated.

Forest plots are also included for most of the cancers. These are generally based on the smoking-adjusted analyses, with the estimates split by region and shown with cohort data first, then case-control, presented in order of publication year.

### Sensitivity analysis

For each estimate included, the value of Q^2 ^is calculated by *w *(*x *- )^2^, where *w *is the inverse-variance weight, *x *is the logarithm of the effect size and  its mean. Q^2 ^is the contribution of the estimate to the heterogeneity chi-squared statistic [116]. Where there is significant (*P *< 0.05) heterogeneity of estimates, sensitivity to potentially outlying estimates is tested by removing that with the largest Q^2 ^value and rerunning the analyses. This process is continued until there is no longer significant heterogeneity.

Sensitivity to the criterion for including estimates based on ST exposure is also tested by rerunning the meta-analyses with the preference list for ST exposure changed from ever use/unspecified use/current use/former use to current use/ever use/unspecified use/former use.

### Meta-regression analysis

For oropharyngeal cancer, fixed-effects regression analysis is used to investigate how the estimates selected for the first set of meta-analyses vary by region (USA; Scandinavia; other), period × study type (cohort; case-control published before 1990; case-control published after 1990), sex (male; female; combined), ST exposure (ever or unspecified use; current use), smoking (any, adjusted for smoking; any, unadjusted for smoking; never) and alcohol adjustment (yes; no). For those other cancers where more than five estimates are available and where there was evidence of significant (*P *< 0.05) heterogeneity, the meta-regression analyses use a more limited variable list: region, sex, and smoking as above, and also study type (cohort; case-control).

Regression analyses are only conducted based on the overall data and smoking-adjusted data. The analyses successively introduce the most significant factor into the model, stopping when no further factor significant at *P *< 0.05 can be added. Significance is estimated by treating the ratio of the deviance per degree of freedom (d.f.) explained by the factor to the residual deviance per d.f. as an F statistic. For oropharyngeal cancer some additional analyses investigate the drop in deviance resulting from introducing each factor individually, and others are conducted having excluded 'outlying' observations with a very high Q^2 ^value.

### Estimating deaths attributable to smoking

RRs for current and former cigarette smokers (compared with never cigarette smokers) for men aged 35+ for seven major cancers caused by smoking (lip/oral cavity/pharynx, oesophagus, pancreas, larynx, lung, bladder, kidney/other urinary organs) were obtained from the American Cancer Society Cancer Prevention Study II (CPS-II) [122]. Numbers of deaths for these seven cancers occurring in US men aged 35+ in 2005 were obtained from WHO [123]. Estimates of the proportion of current and former cigarette smokers in US men aged 35+ in 2005 were obtained from the National Health Interview Survey [124].

Defining D_i _as the number of deaths for cancer i (i = 1,..., 7), R_ci _and R_fi _as the RRs for current and former cigarette smokers for cancer i, and p_c _and p_f _as the proportions of current and former cigarette smokers in the population, the estimated number of deaths, , that would have occurred had the whole population the risk of never smokers, is then estimated by:

The number of deaths avoided from these seven cancers, had the whole population the risk of never smokers (that is, the deaths attributable to smoking) is then estimated by:

### Estimating deaths attributable to ST in a population of never smokers

Let us further define R_si _as the estimated relative risk from ST for cancer i based on the meta-analyses using smoking-adjusted effect estimates. Where R_si _is estimated to be less than 1, it is taken to be 1 for the purposes of calculating deaths attributable to ST.

For a population of never smokers, the number of deaths from cancer i that would have occurred had the same proportion of men used ST as had ever smoked is then estimated by:

The increase in overall deaths from these seven cancers is then given by:

I_1 _can then be compared with E as an indicator of the relative effects of ST and smoking.

Also for a population of never smokers, the number of deaths from cancer i that would have occurred had all the men used ST, is estimated by:

The increase, compared with E, is then calculated by:

## Results

**Figure 1:**
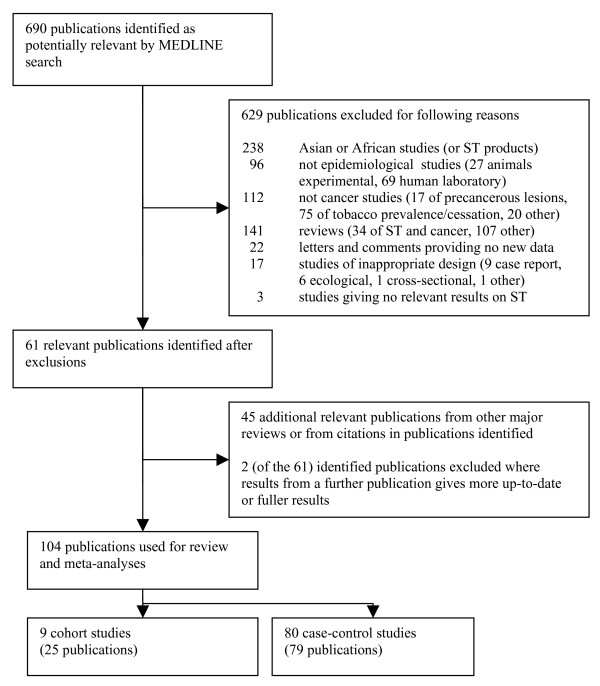
**Flow chart for search strategy for review of literature on smokeless tobacco and cancer**. The flow chart shows the number of publications identified by the MEDLINE search, and the number excluded by reason. The number of additional publications identified from reviews and secondary references is also indicated, as is the total number of publications considered in the review and meta-analysis, subdivided by study type.

The MEDLINE search identified 690 publications. Two hundred and thirty-eight were rejected as describing studies conducted in Asia or Africa or relating to products typically used there, 96 as not describing epidemiological studies, 112 as not relating to cancer and 163 as being reviews, letters or comments not providing primary data. Seventeen were rejected as having an inappropriate study design and three as not providing relevant results. This left 61 apparently relevant publications. Taking into account also citations in recent reviews [1-6,10], and eliminating publications that referred to studies more recently or completely covered in other publications, a total of 104 publications were considered. Twenty-five related to nine cohort studies, and 79 to 80 case-control studies. Fuller details of the search are given in Figure 1, whilst the studies and publications considered are presented in the following two sections.

### Cohort studies

Results relating ST use to mortality or incidence have been reported for nine cohort studies, with results provided by multiple publications for some studies. Six studies have been conducted in the USA and are based on the Lutheran Brotherhood cohort [11-14], the US Veterans cohort [15-19], the Iowa cohort [20], the First National Health and Nutrition Examination Survey (NHANES I) Follow-up cohort [21,22], and the American Cancer Society Cancer Prevention Study I (CPS-I) [23] and Study II (CPS-II) [23-25]. One study was based on two Norway cohorts [14,26,27] while the remaining two were conducted in Sweden; one based on construction workers [28-34], and the other on a cohort in Uppsala County [35]. Fuller details of these studies are given in Table 1. A number of these studies (US Veterans, CPS-I, CPS-II, Swedish Construction Workers) are extremely large, involving at least 100,000 subjects, though the number of ST users is less than this, particularly in the CPS-I and CPS-II studies where the analyses of Henley *et al*. [23] restricted attention to never smokers of cigarettes. The US studies generally present results for combined ST use, the main exception being the analyses of CPS-II [23] where some separate analyses are presented for snuff and chewing tobacco. The results from the Swedish studies relate to snuff use, as do the main results from the Norwegian study [26].

### Case-control studies

Results relating ST use to cancer have been reported for 80 case-control studies, with Table 2 providing details for each study, in chronological order of publication, of the location, period and controls, as well as the exposures, cancer types and sexes studied. Eighteen were published between 1920 and 1975 [36-52], 30 between 1976 and 1990 [53-82] and 32 between 1991 and 2007 [83-114]. In general there was one publication per study, but Bjelke [52] reported results from two studies, while the reference to Gross *et al*. [91] is to a review, which cites results from an unpublished study by Perry *et al*. Of the 80 studies, 56 were conducted in the USA, 11 in Sweden, three in each of Canada, Denmark and the UK, and one in each of Brazil, Norway and Puerto Rico, with one study conducted in five countries. Most of the studies involve only one or a small number of cancer types, but one study [55] involves a very wide range. The majority of the studies involve less than 1,000 cancer cases but 10 are larger than this [38,55,56,66,70,74,89,97,99,106]. The numbers of cancers in ST-exposed subjects are typically much lower than this, as will become evident when the results for the individual sites are presented. Of the different cancer sites, oral cancer is by far the most often studied. Of the 56 US studies, 11 provide results only for chewing tobacco, five only for snuff, and 18 only for ST, with the remaining 22 results for more than one type. Seven of the 11 studies in Sweden restricted attention to snuff, with three also considering chewing and one only considering chewing.

### Adjustment for smoking

ST use is not a major subject for many of the publications from which results have been extracted. While reference is made to ST in the title of one or more papers relating to six of the nine cohort studies (NHANES I, CPS-I, CPS-II, Norway Cohorts, Swedish Construction Workers and Uppsala County), the same is true for only 15 of the 80 case-control studies. For many of the other studies [39,42,59,61,66,70,89,91,102,104,108,109,111,113,114], the reports only provide limited information about ST use in the text, simply giving percentages of users in the cases and controls or even saying there was an association or no association, but without giving supportive data. Many papers consider ST independently of smoking, with no attempt to adjust ST effect estimates for smoking, even though for many of the cancers considered smoking is known to be a cause, and often a major cause.

To summarise the extent to which the available effect estimates were adjusted for smoking, the studies were divided into five groups (A = no information, B = no adjustment, C = never smokers, D = some adjustment, E = more adjustment) as described more fully in the methods. Of the nine cohort studies, the numbers in the five categories were, respectively, 0, 1, 3, 3 and 2. The Iowa study [20] failed to take smoking into account at all, while the CPS-I and CPS-II studies [23] and the main results from NHANES I [22] were restricted to never smokers. In the remaining five cohort studies, the extent of smoking adjustment varied from publication to publication, but amount smoked or duration of smoking were never taken into account in the US Veterans, Norway cohorts and Uppsala County studies so they are classified as group D. In the Lutheran Brotherhood study, amount smoked was taken into account in the analyses of pancreatic cancer [13] and stomach cancer [12], and in the Swedish Construction Workers study, amount smoked was adjusted for in the analyses of stomach and oesophageal cancer [34], and cutaneous squamous cell carcinoma [29], and they are therefore classified as group E.

Of the 80 case-control studies considered, details of the adjustment factors used are not provided in either of the studies reported by Bjelke [52] or in two other studies [93,109] (category A). For a further 38 studies [36,38-40,42,44-46,49,51,53,54,56,57,59,60,63,64,67,70-73,75,78,80-82,84-86,92,95,96,98,100,103,110] the results available for ST are for the whole population, with no adjustment for smoking (category B). In 14 studies [43,47,48,66,69,74,76,83,87,88,99,101,111,112] the only relevant smoking-adjusted results reported are for never smokers (category C). In the remaining 24 studies, some smoking-adjusted results are available for the whole population. Fourteen of these [37,41,50,58,61,62,65,94,97,102,104,107,108,114] can be classified into category D. In only 10 reports [55,68,77,79,89-91,105,106,113], is some account taken of daily dose and/or duration of smoking (category E).

### Oropharyngeal cancer

Table 3 presents individual effect estimates from six cohort and 34 case-control studies, with 36 of the 40 studies providing estimates with CI that could be used in meta-analyses, the other four [40,71,86,92] finding no significant relationship. Thirty-eight of the 41 estimates included in the first meta-analysis (see Table 4) are those given in our earlier review of ST and oral cancer [4], three recently published studies [32,35,113] being introduced into the current analysis. The overall data show an association with any ST use (1.79, 1.36–2.36) that, though highly significant, is based on an extremely heterogeneous set of estimates (*P *< 0.001). Limiting consideration to smoking-adjusted data, the estimate reduces substantially, to 1.36 (1.04–1.77, *n *= 19), though it is still significant, and marked heterogeneity remains (*P *< 0.001). Further limiting attention to estimates adjusted for both smoking and alcohol, the two major risk factors for oropharyngeal cancer [7,8], eliminates both heterogeneity and excess risk (1.07, 0.84–1.37, *n *= 10). A significant relationship is seen in never smokers (1.72, 1.01–2.94, *n *= 9), though the estimates are heterogeneous (*P *= 0.044), and generally based on a very small number of oropharyngeal cancer cases that used ST.

**Table 4:** Oropharyngeal cancer; meta-analysis results

				Heterogeneity		
				
Type of ST (region)^a^	Adjustments/restrictions^b^	Number of estimates (RR/OR ids)^c^	Random-effects RR/OR (95% CI)	χ^2^	I^2^	*P*(χ^2^)
Any	Overall data	*n *= 41 (1, 2, 3, 6, 7, 11, 15, 16, 18, 19, 20, 21, 22, 23, 24, 25, 26, 28, 29, 30, 33, 34, 35, 38, 39, 41, 42, 46, 47, 48, 49, 51, 55, 56, 58, 60, 63, 65, 73, 74, 77)	1.79 (1.36–2.36)	335.6	88.1	< 0.001
	Smoking-adjusted	*n *= 19 (2, 3, 6, 7, 11, 13, 18, 26, 35, 43, 48, 51, 55, 56, 58, 63, 69, 74, 77)	1.36 (1.04–1.77)	69.5	74.1	< 0.001
	Smoking and alcohol adjusted	*n *= 10 (2, 3, 11, 51, 55, 56, 63, 69, 74, 77)	1.07 (0.84–1.37)	12.5	28.0	0.186
	Never smokers	*n *= 9 (2, 3, 10, 12, 27, 43, 48, 59, 72)	1.72 (1.01–2.94)	15.9	49.7	0.044
	Never smokers – alcohol adjusted	*n *= 3 (2, 3, 12)	1.87 (0.82–4.27)	0.6	0.0	0.731
						
Any (USA)^d^	Overall data	*n *= 31 (1, 2, 3, 15, 16, 18, 19, 20, 21, 22, 23, 24, 25, 26, 29, 30, 33, 34, 35, 38, 39, 41, 42, 46, 49, 51, 55, 56, 58, 65, 74)	2.16 (1.55–3.02)	275.8	89.1	< 0.001
	Smoking-adjusted	*n *= 12 (2, 3, 13, 18, 26, 35, 43, 51, 55, 56, 58, 74)	1.65 (1.22–2.25)	33.6	67.3	< 0.001
	Smoking and alcohol adjusted	*n *= 6 (2, 3, 51, 55, 56, 74)	1.04 (0.80–1.35)	1.8	0.0	0.875
	Never smokers	*n *= 5 (2, 3, 27, 43, 59)	3.33 (1.76–6.32)	3.5	0.0	0.476
	Never smokers – alcohol adjusted	*n *= 2 (2, 3)	1.58 (0.52–4.81)	0.4	0.0	0.512
						
Snuff (Scandinavia)	Overall data	*n *= 7 (6, 7, 11, 48, 63, 69, 77)	0.97 (0.68–1.37)	14.5	58.8	0.024
	Smoking-adjusted	*n *= 7 (6, 7, 11, 48, 63, 69, 77)	0.97 (0.68–1.37)	14.5	58.8	0.024
	Smoking and alcohol adjusted	*n *= 4 (11, 63, 69, 77)	1.10 (0.64–1.90)	10.7	71.9	0.014
	Never smokers	*n *= 4 (10, 12, 48, 72)	1.01 (0.71–1.45)	2.2	0.0	0.524
	Never smokers – alcohol adjusted	*n *= 1 (12)	2.30 (0.67–7.92)	--	--	--
						
Published since 1990	Overall data	*n *= 18 (1, 2, 3, 6, 7, 11, 48, 49, 51, 55, 56, 58, 60, 63, 65, 73, 74, 77)	1.28 (0.94–1.76)	81.7	79.2	< 0.001
	Smoking-adjusted	*n *= 14 (2, 3, 6, 7, 11, 48, 51, 55, 56, 58, 63, 69, 74, 77)	1.00 (0.83–1.20)	18.5	29.8	0.139
	Smoking and alcohol adjusted	*n *= 10 (2, 3, 11, 51, 55, 56, 63, 69, 74, 77)	1.07 (0.84–1.37)	12.5	28.0	0.186
	Never smokers	*n *= 7 (2, 3, 10, 12, 48, 59, 72)	1.24 (0.80–1.90)	7.5	20.1	0.277
	Never smokers – alcohol adjusted	*n *= 3 (2, 3, 12)	1.87 (0.82–4.27)	0.6	0.0	0.731

^a ^For each study/sex, the RR/OR for ST from Table 3 was included if available, otherwise that for chewing tobacco or snuff was used.

^b ^Smoking-adjusted includes estimates for smokers and non-smokers combined, adjusted for smoking if available, and estimates for never smokers otherwise.

^c ^The actual estimates included are identified by their RR/OR identification numbers as given in Table 3.

^d ^Includes estimates 24 and 25 from a study in Puerto Rico [49].

CI = confidence interval; ST = smokeless tobacco; OR = odds ratio; RR = relative risk.

When the analyses are restricted to US studies, the pattern is similar to that for the overall data, with the effect estimates reduced when attention is limited to those that are smoking-adjusted, and close to 1.0 when estimates that are adjusted both for smoking and alcohol are considered. The effect estimate for never smokers is significantly increased (3.33, 1.76–6.32), based on five small studies, in total involving 19 ST-exposed oropharyngeal cancer cases.

No real evidence of a relationship with snuff use is seen in studies conducted in Scandinavia, where seven estimates, all adjusted for smoking, and four additionally adjusted for alcohol, give a combined estimate of 0.97 (0.68–1.37). However some heterogeneity should be noted, a high RR of 3.1 (1.5–6.6) in the Uppsala County study [35] conflicting with six other estimates ranging from 0.67 to 1.10.

Many of the higher estimates seen in Table 4 come from older studies which often did not adjust for smoking. If attention is limited to studies published since 1990, which generally did adjust, no association is seen. Indeed, the combined estimate from the 14 smoking-adjusted studies published since 1990 is 1.00 (0.83–1.20), and shows no significant heterogeneity.

**Figure 2:**
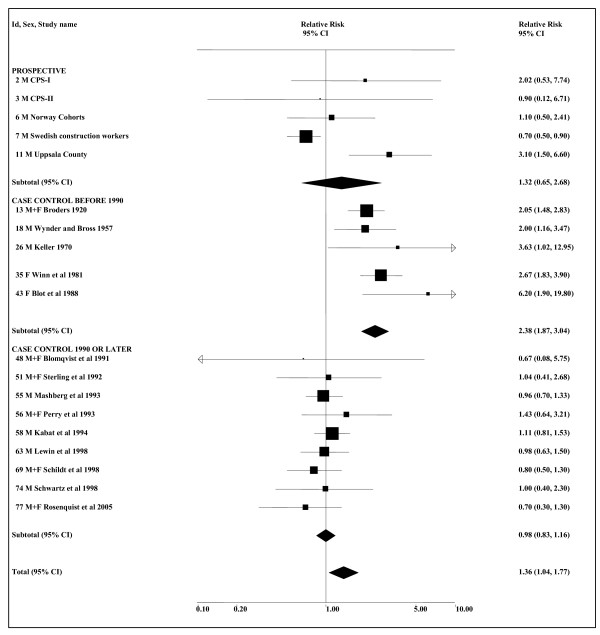
**Smokeless tobacco and oropharyngeal cancer by study type and period of publication (smoking-adjusted data)**. The 19 individual smoking-adjusted relative risk (RR) and 95% confidence interval (CI) estimates separated by study type, and for case-control studies by period of publication, are shown numerically and also graphically on a logarithmic scale. They are sorted in order of year of publication. In the graphical representation individual RR estimates are indicated by a solid square, with the area of the square proportional to the weight (inverse-variance) of the estimate. Also shown are the combined estimates, for the subgroups and overall, derived by random-effects meta-analysis. These are represented by a diamond of standard height, with the width indicating the 95% CI. See Table 3 for further details relating to the estimates, and Table 4 for fuller details of the meta-analyses.

While the choice of 1990 as the cut-point was not defined *a priori*, the change in estimates about that time is very clear. As shown in Figure 2, smoking-adjusted estimates for case-control studies published between 1920 and 1988 are consistently high (overall 2.38, 95% CI 1.87–3.04), while estimates for case-control studies published between 1991 and 2005 show no association at all (0.98, 0.83–1.16). There is no evidence of heterogeneity within either period (*P *= 0.34 for pre-1990 and *P *= 0.93 for post-1990) and a highly significant (*P *< 0.001) difference between estimates in the two periods. Smoking-adjusted estimates for the cohort studies which, though published between 2005 and 2008, generally cover a long follow-up period extending from before 1990, give an intermediate result (1.32, 0.65–2.68).

The findings are very similar to those in an earlier review [4]. That review provides additional meta-analyses of the slightly smaller data set, further investigating variation by type of ST, sex, study design, study location and study period. It also provides full details of the various types of cancer that have been considered in the source papers.

The evidence presented suggests that snuff as used in Scandinavia has no effect on oropharyngeal cancer risk. Products used in the past in the USA may have increased the risk but any effect that exists now seems likely to be quite small.

### Oesophageal cancer

Table 5 summarises the data from four cohort and 10 case-control studies. For five of these studies effect estimates with CI are not available, one of these [52] reporting a 'synergistic effect of tobacco chewing and alcohol', another [19] presenting a RR of 2.28, but not whether it was significant, and the others [14,40,60] showing no significant relationship. Of the remaining nine studies, six provide smoking-adjusted estimates, three of which are also adjusted for alcohol. Though estimates are generally somewhat above 1.0 in these nine studies, they are rarely significant, exceptions being the estimate of 1.92 (1.00–3.68) for snuff in never smokers in the Swedish Construction Workers study [34] and that for chewing of 2.39 (1.23–4.64) in the Wynder and Bross case-control study [44].

**Table 5:** Oesophageal cancer; individual effect (relative risk/odds ratio) estimates

	ST use				RR/OR			
				
Source^a^	Type^b^	Exposure^c^	Smoking	Sex^d^	Id.	Cases^e^	Estimate (95%CI)^d^	Adjustment factors^f^
**Cohort studies**								
								
Lutheran Brotherhood: IARC Monograph 37 1985 [14]	ST	Ever	Any	M	1	NA	2.6 (not significant)	age, res
US Veterans: Winn *et al*. 1982 [19]	ST	Ever	Never	M^g^	2	1	2.28 (NA)	age
Norway cohorts: Boffetta *et al*. 2005 [26]	Snuff	Current	Any	M	3	4	1.06 (0.35–3.23)	age, smok
		Former		M	4	5	1.90 (0.69–5.27)	
		Ever		M	5	9	1.40 (0.61–3.24)	
Swedish construction workers: Zendehdel *et al*. 2008 [34]	Snuff	Ever	Any	M	6	77	1.00 (0.79–1.27)^h^	age, bmi, smok
			Never		7	11	1.92 (1.00–3.68)^i^	age, bmi
								
**Case-control studies**								
Wynder *et al*. 1957 [40]	Chew	Ever	Any	M	8	NA	no association^j^	none
Wynder and Bross 1961 [44]	Chew	Ever	Any	M	9	21	2.39 (1.23–4.64)^k^	none
Martinez *et al*. 1969 [49]	Chew	Use	Never	M	10	3	1.18 (0.28–4.90)^k^	none
				F	11	7	2.69 (0.92–7.87)^k^	
Bjelke *et al*. 1974 USA [52]	Chew	Use	NA	NA	12	NA	association^l^	NA
Williams and Horm 1977 [55]	ST	Ever	Any	M	13	2	0.55 (0.13–2.31)	none
Wynder and Stellman 1977 [56]	Chew	Ever	Any	M	14	20	1.23 (0.76–1.99)^k^	none
	Snuff				15	8	1.65 (0.78–3.49)^k^	
	ST				16	28	1.35 (0.89–2.06)^m^	
Pottern *et al*. 1981 [60]	Chew	Ever	Any	M	17	4	no association^n^	none
	Snuff				18	2	no association^n^	
Morris Brown *et al*. 1988 [76]	ST	Ever	Never	M	19	1	1.20 (0.10–13.30)	alc, incm
Lewin *et al*. 1998 [102]	Snuff	Current	Any	M	20	10	1.10 (0.50–2.40)	age, alc, res, smok
		Former			21	9	1.30 (0.60–3.10)	
		Ever			22	19	1.20 (0.70–2.20)	
Lagergren *et al*. 2000 [108]	Snuff	Ever	Any	M+F	23	68	1.31 (0.89–1.92)^k^	age, alc, bmi, diet, edu, exer, rflx, sex, smok

^a ^Fuller details of the studies are given in Tables 1 and 2.

^b ^ST implies smokeless tobacco unspecified, or combined snuff use or chewing.

^c ^Ever, former and current ST use were compared with never ST. Use indicates timing not given and comparison is with non-use.

^d ^NA = not available.

^e ^'Id.' is the RR/OR identification number used in Table 6, and 'Cases' is the number of cases in ST users as defined. NA = not available.

^f ^Abbreviations used: alc = alcohol, bmi = body mass index, edu = education, exer = exercise, incm = incidence or mortality, res = area of residence, rflx = reflux symptoms, smok = smoking, NA = not available.

^g ^The population included < 0.5% females.

^h ^RRs for adenocarcinoma (1.0, 95% CI 0.6–1.5) and squamous cell carcinoma (1.0, 0.8–1.4) combined.

^i ^RRs for adenocarcinoma (0.2, 95% CI 0.0–1.9) and squamous cell carcinoma (3.5, 1.6–7.6) combined.

^j ^The average ridit duration of chewing was non-significantly lower in the oesophageal cancer cases.

^k ^RR/OR and/or 95% CI estimated from data provided in the source.

^l ^The abstract noted a "synergistic effect of tobacco chewing and alcohol".

^m ^RR/OR and/or 95% CI estimated from data provided in the source assuming that no one both chewed and used snuff.

^n ^The authors noted the percentage of ever users was "slightly higher" in the controls than in the cases for chewing but not for snuff.

CI = confidence interval; ST = smokeless tobacco; OR = odds ratio; RR = relative risk.

**Figure 3:**
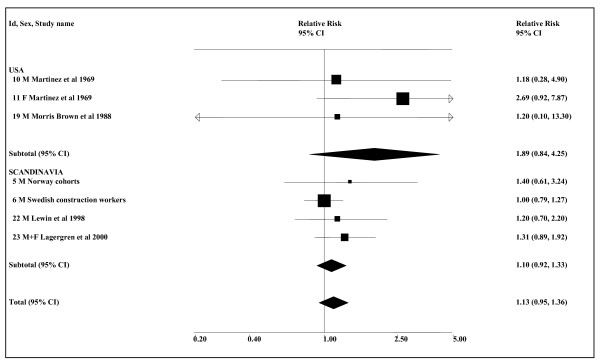
**Smokeless tobacco and oesophageal cancer by region (smoking-adjusted data)**. The seven individual smoking-adjusted relative risk (RR) and 95% confidence interval (CI) estimates, separated by region, are shown numerically and also graphically on a logarithmic scale. They are sorted in order of year of publication within study type (cohort, case-control). In the graphical representation individual RR estimates are indicated by a solid square, with the area of the square proportional to the weight (inverse-variance) of the estimate. Also shown are the combined estimates, for the subgroups and overall, derived by random-effects meta-analysis. These are represented by a diamond of standard height, with the width indicating the 95% CI. See Table 5 for further details relating to the estimates, and Table 6 for fuller details of the meta-analyses.

The meta-analyses (see Table 6 and Figure 3) show some indication of an association, though this is not always statistically significant. Based on all available smoking-adjusted data, the combined estimate for any ST use is 1.13 (0.95–1.36, *n *= 7), somewhat lower than when there is no restriction to smoking-adjusted data (1.25, 1.03–1.51, *n *= 10). The corresponding analyses show no real indication of an effect for snuff in Scandinavia, but are more suggestive for the USA. Even here, the smoking-adjusted estimate is not significant (1.89, 0.84–4.25), though this is based on only three small studies, involving a total of 11 cases using ST. The estimates based on all the available smoking-adjusted data include an any smoking RR of 1.00 (0.79–1.27) from the study with the largest weight, the Swedish Construction Workers study [34], this RR being derived by combining the findings for adenocarcinoma and squamous cell carcinoma. The meta-analyses for never smokers give a higher combined estimate of 1.91 (1.15–3.17, *n *= 4) for any ST use, mainly because they use a higher (combined adeno/squamous) estimate of 1.92 (1.00–3.68) for the Swedish Construction Workers study [34].

**Table 6:** Oesophageal cancer; meta-analysis results

				Heterogeneity		
				
Type of ST (region)^a^	Adjustments/restrictions^b^	Number of estimates (RR/OR ids)^c^	Random-effects RR/OR (95% CI)	χ^2^	I^2^	*P*(χ^2^)
Any	Overall data	10 (5, 6, 9, 10, 11, 13, 16, 19, 22, 23)	1.25 (1.03–1.51)	10.3	13.0	0.324
	Smoking-adjusted	7 (5, 6, 10, 11, 19, 22, 23)	1.13 (0.95–1.36)	4.4	0.0	0.623
	Never smokers	4 (7, 10, 11, 19)	1.91 (1.15–3.17)	1.0	0.0	0.810
						
Any (USA)^d^	Overall data	6 (9, 10, 11, 13, 16, 19)	1.56 (1.11–2.19)	5.2	4.6	0.387
	Smoking-adjusted	3 (10, 11, 19)	1.89 (0.84–4.25)	1.0	0.0	0.617
	Never smokers	3 (10, 11, 19)	1.89 (0.84–4.25)	1.0	0.0	0.617
						
Snuff (Scandinavia)	Overall data	4 (5, 6, 22, 23)	1.10 (0.92–1.33)	1.8	0.0	0.61
	Smoking-adjusted	4 (5, 6, 22, 23)	1.10 (0.92–1.33)	1.8	0.0	0.61
	Never smokers	1 (7)	1.92 (1.00–3.68)	--	--	--

^a ^For each study/sex, the RR/OR for ST from Table 5 was included if available, otherwise that for chewing tobacco or snuff was used.

^b ^Smoking-adjusted includes estimates for smokers and non-smokers combined, adjusted for smoking if available, and estimates for never smokers otherwise.

^c ^The actual estimates included are identified by their RR/OR identification numbers as given in Table 5.

^d ^Includes estimates 10 and 11 from a study in Puerto Rico [49]

CI = confidence interval; ST = smokeless tobacco; OR = odds ratio; RR = relative risk.

Overall, the data must be regarded as providing suggestive evidence of a possible weak relationship between ST use and oesophageal cancer.

### Stomach cancer

**Figure 4:**
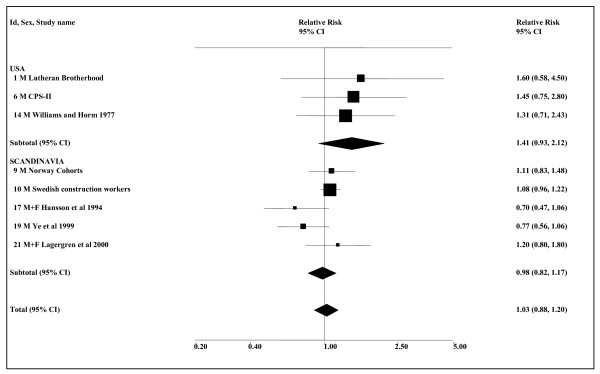
**Smokeless tobacco and stomach cancer by region (smoking-adjusted data)**. The eight individual smoking-adjusted relative risk (RR) and 95% confidence interval (CI) estimates, separated by region, are shown numerically and also graphically on a logarithmic scale. They are sorted in order of year of publication within study type (cohort, case-control). In the graphical representation individual RR estimates are indicated by a solid square, with the area of the square proportional to the weight (inverse-variance) of the estimate. Also shown are the combined estimates, for the subgroups and overall, derived by random-effects meta-analysis. These are represented by a diamond of standard height, with the width indicating the 95% CI. See Table 7 for further details relating to the estimates, and Table 8 for fuller details of the meta-analyses.

Table 7 presents results from 12 studies, eight of which provide a total of 17 estimates which could be used in meta-analyses. Although the Swedish construction workers study [34] shows a significant increase in risk of stomach cancer associated with snuff use for never smokers (RR 1.33, 95% CI 1.03–1.72), no other significant associations are reported, and the meta-analyses conducted (see Table 8 and Figure 4) are all non-significant. Based on smoking-adjusted estimates from eight studies, the combined RR estimate is 1.03 (95% CI 0.88–1.20). Four studies did not provide detailed data. No association with stomach cancer was reported by Weinberg *et al*. [67] or for the US data considered by Bjelke [52]. However, Bjelke did report an "Association ... with tobacco chewing" for the Norwegian data, and a standardised mortality ratio of 1.51 was given for the US Veterans' Study [19], but not whether this was statistically significant.

**Table 7:** Stomach cancer; individual effect (relative risk/odds ratio) estimates

	ST use				RR/OR			
				
Source^a^	Type^b^	Exposure^c^	Smoking	Sex^d^	Id.	Cases^e^	Estimate (95%CI)^d^	Adjustment factors^f^
**Cohort studies**								
								
Lutheran Brotherhood: Kneller *et al*. 1991 [12]	ST	Ever	Any	M	1	18	1.60 (0.58–4.50)	age, byr, smok
			Never	M	2	3	3.80 (1.00–14.32)	age, byr
US Veterans: Winn *et al*. 1982 [19]	ST	Ever	Never	M^g^	3	NA	1.51 (NA)	age
CPS-II: Chao *et al*. 2002 [24]	ST	Current	Never	M	4	8	1.58 (0.76–3.28)	age, asp, diet, edu, fhis, race, vit
		Former			5	2	1.11 (0.27–4.50)	
		Ever			6	10	1.45 (0.75–2.80)^h^	
Norway cohorts: Boffetta *et al*. 2005 [26]	Snuff	Current	Any	M	7	42	1.00 (0.71–1.42)	age, smok
		Former			8	32	1.29 (0.87–1.91)	
		Ever			9	74	1.11 (0.83–1.48)	
Swedish construction workers: Zendehdel *et al*. 2008 [34]	Snuff	Ever	Any	M	10	311	1.08 (0.96–1.22)^i^	age, bmi, smok
		Ever	Never	M	11	76	1.33 (1.03–1.72)^j^	age, bmi
								
**Case-control studies**								
								
Bjelke 1974 (USA) [52]	Chew	Use	Any	NA	12	NA	no association	NA
Bjelke 1974 (Norway) [52]	Chew	Use	Any	NA	13	NA	association	NA
Williams and Horm 1977 [55]	ST	Ever	Any	M	14	12	1.31 (0.71–2.43)^h^	age, race, smok
				F	15	2	1.50 (0.36–6.26)	none
Weinberg *et al*. 1985 [67]	Chew	Ever	Any	M	16	NA	no association	none
Hansson *et al*. 1994 [94]	Snuff	Use	Any	M+F	17	NA	0.70 (0.47–1.06)	age, ses, sex, smok
Ye *et al*. 1999 [107]	Chew	Ever	Any	M+F	18	8	1.30 (0.54–3.12)^h^	none
	Snuff	Ever		M	19	83	0.77 (0.56–1.06)^h^	age, alc, bmi, res, ses, smok
			Never	M	20	11	0.50 (0.20–1.20)	age, alc, bmi, res, ses
Lagergren *et al*. 2000 [108]	Snuff	Ever	Any	M+F	21	53	1.20 (0.80–1.80)	age, alc, bmi, diet, edu, exer, rflx, sex, smok

^a ^Fuller details of the studies are given in Tables 1 and 2.

^b ^ST implies smokeless tobacco unspecified, or combined snuff use or chewing.

^c ^Ever, former and current ST use were compared with never ST. Use indicates timing not given and comparison is with non use.

^d ^NA = not available.

^e ^'Id.' is the RR/OR identification number used in Table 8, and 'Cases' is the number of cases in ST users as defined. NA = not available.

^f ^Abbreviations used: alc = alcohol, asp = aspirin, bmi = body mass index, byr = birth year, edu = education, exer = exercise, fhis = family history of stomach cancer, incm = incidence or mortality, res = area of residence, rflx = reflux symptoms, ses = socioeconomic status, smok = smoking, vit = vitamins, NA = not available.

^g ^The population included < 0.5% females.

^h ^Estimated from data provided in the source.

^i ^RRs for cardia (1.0, 95% CI 0.8–1.4) and noncardia stomach cancer (1.1, 1.0–1.3) combined.

^j ^RRs for cardia (0.9, 95% CI 0.4–2.0) and noncardia stomach cancer (1.4, 1.1–1.9) combined.

CI = confidence interval; ST = smokeless tobacco; OR = odds ratio; RR = relative risk.

**Table 8:** Stomach cancer; meta-analysis results

				Heterogeneity		
				
Type of ST (region)^a^	Adjustments/restrictions^b^	Number of estimates (RR/OR ids)^c^	Random-effects RR/OR (95% CI)	χ^2^	I^2^	*P*(χ^2^)
Any	Overall data	9 (1, 6, 9, 10, 14, 15, 17, 19, 21)	1.03 (0.90–1.19)	10.5	24.0	0.230
	Smoking-adjusted	8 (1, 6, 9, 10, 14, 17, 19, 21)	1.03 (0.88–1.20)	10.3	31.9	0.173
	Never smokers	4 (2, 6, 11, 20)	1.27 (0.75–2.13)	7.0	57.2	0.072
						
Any (USA)	Overall data	4 (1, 6, 14, 15)	1.41 (0.95–2.10)	0.1	0.0	0.988
	Smoking-adjusted	3 (1, 6, 14)	1.41 (0.93–2.12)	0.1	0.0	0.942
	Never smokers	2 (2, 6)	1.96 (0.82–4.70)	1.6	38.2	0.203
						
Snuff (Scandinavia)	Overall data	5 (9, 10, 17, 19, 21)	0.98 (0.82–1.17)	8.1	50.4	0.089
	Smoking-adjusted	5 (9, 10, 17, 19, 21)	0.98 (0.82–1.17)	8.1	50.4	0.089
	Never smokers	2 (11, 20)	0.90 (0.35–2.30)	4.2	76.4	0.040

^a ^For each study/sex, the RR/OR for ST from Table 7 was included if available, otherwise that for chewing tobacco or snuff was used.

^b ^Smoking-adjusted includes estimates for smokers and non-smokers combined, adjusted for smoking if available, and estimates for never smokers otherwise.

^c ^The actual estimates included are identified by their RR/OR identification numbers as given in Table 7.

CI = confidence interval; ST = smokeless tobacco; OR = odds ratio; RR = relative risk.

The combined evidence does not indicate an effect of ST use on the risk of stomach cancer.

### Pancreatic cancer

**Figure 5:**
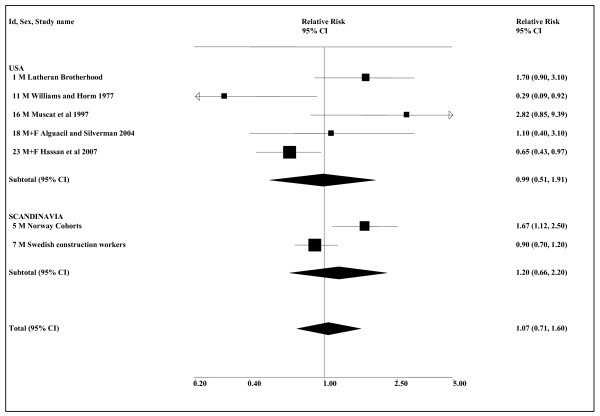
**Smokeless tobacco and pancreatic cancer by region (smoking-adjusted data)**. The seven individual smoking-adjusted relative risk (RR) and 95% confidence interval (CI) estimates, separated by region, are shown numerically and also graphically on a logarithmic scale. They are sorted in order of year of publication within study type (cohort, case-control). In the graphical representation individual RR estimates are indicated by a solid square, with the area of the square proportional to the weight (inverse-variance) of the estimate. Also shown are the combined estimates, for the subgroups and overall, derived by random-effects meta-analysis. These are represented by a diamond of standard height, with the width indicating the 95% CI. See Table 9 for further details relating to the estimates, and Table 10 for fuller details of the meta-analyses.

Table 9 presents results from four cohort and seven case-control studies. For four of the studies effect estimates that can be included in meta-analyses are not available; two [75,84] of these studies merely reported finding no association, one [19] reported an elevated RR of 1.65 with no CI, and another [82] a reduced RR of 0.80, also with no CI. Of the other seven studies, significant increases have been reported in two. The Norway cohorts study [26] reports an increase in ever users of snuff in a smoking-adjusted analysis based on the whole population (1.67, 95% CI 1.12–2.50) but not in an analysis based on never smokers (0.85, 0.24–3.07). Conversely, the Swedish construction workers study shows no increase in a smoking-adjusted analysis based on the whole population (0.9, 0.7–1.2), but an increase in never smokers (2.0, 1.2–3.3). None of the three meta-analyses presented in Table 10 (see also Figure 5) for any ST use show any significant increase, though they all show evidence of heterogeneity. Smoking-adjusted overall population effect estimates are available for all seven studies considered, the combined estimate being 1.07 (0.71–1.60). For never smokers, the estimate is 1.23 (0.66–2.31, *n *= 5). No significant associations are seen in the separate meta-analyses for the USA and Scandinavia.

**Table 9:** Pancreatic cancer; individual effect (relative risk/odds ratio) estimates

	ST use				RR/OR			
				
Source^a^	Type^b^	Exposure^c^	Smoking	Sex	Id.	Cases^d^	Estimate (95%CI)^e^	Adjustment factors^f^
**Cohort studies**								
								
Lutheran Brotherhood: Zheng *et al*. 1993 [13]	ST	Ever	Any	M	1	16	1.70 (0.90–3.10)	age, alc, smok
US Veterans: Winn *et al*. 1982 [19]	ST	Ever	Never	M^g^	2	NA	1.65 (NA)	age
Norway cohorts: Boffetta *et al*. 2005 [26]	Snuff	Current	Any	M	3	27	1.60 (1.00–2.55)	age, smok
		Former			4	18	1.80 (1.04–3.09)	
		Ever			5	45	1.67 (1.12–2.50)	
		Ever	Never		6	3	0.85 (0.24–3.07)	age
Swedish construction workers: Luo *et al*. 2007 [32]	Snuff	Ever	Any	M	7	NA	0.90 (0.70–1.20)	age, bmi, smok
		Current	Never		8	18	2.10 (1.20–3.60)	age, bmi
		Former			9	2	1.40 (0.40–5.90)	
		Ever			10	20	2.00 (1.20–3.30)	
								
**Case-control studies**								
								
Williams and Horm 1977[55]	ST	Ever	Any	M	11	3	0.29 (0.09–0.92)^h^	age, race, smok
Falk *et al*. 1988 [75]	Chew	Use	Any	M+F	12	NA	no association	none
	Snuff				13	NA	no association	
Farrow and Davis 1990 [82]	Chew	Ever	Any	M	14	NA	0.80 (NA)	edu, race
Ghadirian *et al*. 1991[84]	Chew	Use	Any	M+F	15	NA	no association	none
Muscat *et al*. 1997 [101]	Chew	Ever	Never^i^	M	16	6	2.82 (0.85–9.39)^j^	none
	Snuff		Any		17	2	1.32 (0.22–7.93)	
Alguacil and Silverman 2004 [111]	ST	Ever	Never^k^	M+F	18	5	1.10 (0.40–3.10)	age, race, res, sex, smok^k^
Hassan *et al*. 2007 [114]	Chew	Ever	Any	M+F	19	34	0.70 (0.40–1.10)	age, alc, diab, edu, mar, race, res, sex, smok
			Never		20	10	0.60 (0.30–1.40)	age, alc, diab, edu, mar, race, res, sex
	Snuff	Ever	Any		21	18	0.60 (0.30–1.10)	age, alc, diab, edu, mar, race, res, sex, smok
			Never		22	4	0.50 (0.10–1.50)	age, alc, diab, edu, mar, race, res, sex
	ST	Ever	Any		23	52	0.65 (0.43–0.97)^l^	age, alc, diab, edu, mar, race, res, sex, smok
			Never		24	14	0.57 (0.29–1.11)^l^	age, alc, diab, edu, mar, race, res, sex

^a ^Fuller details of the studies are given in Tables 1 and 2.

^b ^ST implies smokeless tobacco unspecified, or combined snuff use or chewing.

^c ^Ever, former and current ST use were compared with never ST. Use indicates timing not given and comparison is with non use.

^d ^'Id.' is the RR/OR identification number used in Table 10, and 'Cases' is the number of cases in ST users as defined. NA = not available.

^e ^NA = not available.

^f ^Abbreviations used: alc = alcohol consumption, bmi = body mass index, diab = diabetes, edu = education, mar = marital status, res = area of residence, smok = smoking.

^g ^The population included < 0.5% females.

^h ^RR/OR and/or 95% CI estimated from data provided in the source.

^i ^Includes long-term (10+ years) quitters.

^j ^Personal communication from Dr Muscat. The estimate given in the source of 3.60 (1.00–12.80) is for noncurrent smokers.

^k ^Estimates are for never cigarette smokers with adjustment for other tobacco use.

^l ^RR/OR and/or 95% CI estimated from data provided in the source assuming that no one both chewed and used snuff.

CI = confidence interval; ST = smokeless tobacco; OR = odds ratio; RR = relative risk.

**Table 10:** Pancreatic cancer; meta-analysis results

				Heterogeneity		
				
Type of ST (region)^a^	Adjustments/restrictions^b^	Number of estimates (RR/OR ids)^c^	Random-effects RR/OR (95% CI)	χ^2^	I^2^	*P*(χ^2^)
Any	Overall data	7 (1, 5, 7, 11, 17, 18, 23)	1.00 (0.68–1.47)	18.5	67.5	0.005
	Smoking-adjusted	7 (1, 5, 7, 11, 16, 18, 23)	1.07 (0.71–1.60)	21.2	71.7	0.002
	Never smokers	5 (6, 10, 16, 18, 24)	1.23 (0.66–2.31)	10.7	62.7	0.030
						
Any (USA)	Overall data	5 (1, 11, 17, 18, 23)	0.86 (0.47–1.57)	10.2	61.0	0.037
	Smoking-adjusted	5 (1, 11, 16, 18, 23)	0.99 (0.51–1.91)	13.8	71.0	0.008
	Never smokers	3 (16, 18, 24)	1.09 (0.44–2.67)	5.4	63.0	0.067
						
Snuff (Scandinavia)	Overall data	2 (5, 7)	1.20 (0.66–2.20)	6.3	84.1	0.012
	Smoking-adjusted	2 (5, 7)	1.20 (0.66–2.20)	6.3	84.1	0.012
	Never smokers	2 (6, 10)	1.61 (0.77–3.34)	1.5	33.2	0.221

^a ^For each study/sex, the RR/OR for ST from Table 9 was included if available, otherwise that for chewing tobacco or snuff was used.

^b ^Smoking-adjusted includes estimates for smokers and non-smokers combined, adjusted for smoking if available, and estimates for never smokers otherwise.

^c ^The actual estimates included are identified by their RR/OR identification numbers as given in Table 9.

CI = confidence interval; ST = smokeless tobacco; OR = odds ratio; RR = relative risk.

At most, the overall data weakly suggest a possible effect of ST on pancreatic cancer risk. A fuller discussion of these data is available elsewhere [5].

### Other cancers of the digestive system

Table 11 summarises evidence relating to cancers of the digestive system other than those considered already in Tables 5, 7 and 9. Nine studies are considered, four cohort and five case-control, with one or two studies providing data for colon cancer, rectal cancer, colorectal cancer, small intestine cancer, liver cancer, gall bladder and bile duct cancer. These data, which are insufficient for meta-analysis, include two statistically significant effect estimates: an RR of 1.9 (1.2–3.1) for rectal cancer and ST use from the US Veterans study [18] and a remarkably high OR from the case-control study of Chow *et al*. [93] of 18.0 (1.4–227.7) for bile duct cancer and chewing tobacco, based on only three exposed cases.

**Table 11:** Other cancers of the digestive system; individual effect (relative risk/odds ratio) estimates

	ST use				RR/OR			
				
Source^a^	Type^b^	Exposure^c^	Smoking	Sex^d^	Id.	Cases^e^	Estimate (95%CI)^d^	Adjustment factors^f^
**Cohort studies**								
								
US Veterans: Heineman *et al*. 1995 [18]								
- colon cancer	ST	Ever	Never	M^g^	1	39	1.20 (0.90–1.70)^h^	age, sed, ses, time, yriv
- rectal cancer			Never		2	17	1.90 (1.20–3.10)^h^	
US Veterans: Winn *et al*. 1982 [19]								
- liver cancer	ST	Ever	Never	M^g^	3	NA	2.81 (NA)	age
NHANES I: Accortt *et al*. 2005 [22]								
- digestive cancer	ST	Ever	Never	M	4	13	0.80 (0.40–1.80)	age, pov, race
				F	5	4	0.80 (0.30–2.40)	
CPS-I: Henley *et al*. 2005 [23]								
- digestive cancer	ST	Current	Never	M	6	153	1.26 (1.05–1.52)	age, alc, asp, bmi, diet, edu, exer, occ, race
CPS-II: Henley *et al*. 2005 [23]								
- digestive cancer	ST	Current	Never	M	7	48	1.04 (0.77–1.38)	age, alc, asp, bmi, diet, edu, exer, occ, race
		Former			8	19	0.99 (0.63–1.57)	
		Ever			9	67	1.03 (0.80–1.31)^h^	
								
**Case-control studies**								
Bjelke 1974 [52] USA								
- colorectal cancer	Chew	Use	Any	NA	10	NA	No association	NA
Bjelke 1974 [52] Norway								
- colorectal cancer	Chew	Use	Any	NA	11	NA	No association	NA
Williams and Horm 1977 [55]								
- small intestine cancer	ST	Ever	Any	M	12	2	3.11 (0.65–14.8)^h^	age, race, smok
- colon cancer	ST	Ever	Any	M	13	30	1.36 (0.90–2.07)^h^	age, race, smok
				F	14	7	1.28 (0.58–2.87)^h^	
- rectal cancer	ST	Ever	Any	M	15	13	0.75 (0.42–1.35)^h^	age, race, smok
				F	16	2	0.87 (0.21–3.62)^h^	
- liver cancer	ST	Ever	Any	M	17	1	0.58 (0.08–4.39)^h^	none
- gall bladder cancer	ST	Ever	Any	M	18	1	0.41 (0.05–3.04)^h^	none
Sterling *et al*. 1992 [89]								
- digestive cancer	ST	Ever	Any	M+F	19	555	0.40 (0.24–0.69)^h^	age, alc, occ, race, sex, smok
Chow *et al*. 1994 [93]								
- bile duct cancer^i^	Chew	Use	Any	M	20	3	18.0 (1.40–227.70)	NA

^a ^Fuller details of the studies are given in Tables 1 and 2.

^b ^ST implies smokeless tobacco unspecified, or combined snuff use or chewing.

^c ^Ever, former and current ST use were compared with never ST. Use indicates timing not given and comparison is with non use.

^d ^NA = not available.

^e ^'Id.' is the RR/OR identification number used in Table 12, and 'Cases' is the number of cases in ST users as defined. NA = not available.

^f ^Abbreviations used: alc = alcohol, asp = aspirin, bmi = body mass index, edu = education, exer = exercise, occ = occupation, pov = poverty, sed = sedentary lifestyle, ses = socioeconomic status, smok = smoking, yriv = year of interview, NA = not available.

^g ^The population included < 0.5% females.

^h ^RR/OR and/or 95% CI estimated from data provided in the source.

^i ^Results are for cancer of ampulla of Vater; extrahepatic bile duct cancers were also studied, but results were not given for chewing.

CI = confidence interval; ST = smokeless tobacco; OR = odds ratio; RR = relative risk.

**Figure 6:**
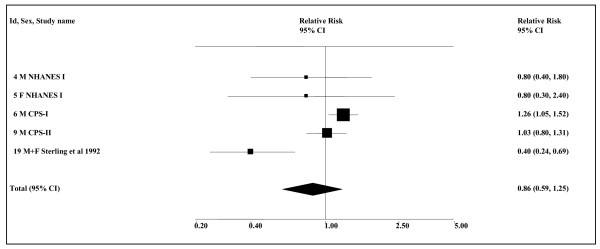
**Smokeless tobacco and overall digestive cancer (USA smoking-adjusted data)**. The five individual relative risk (RR) and 95% confidence interval (CI) estimates, all smoking-adjusted and for the USA, are shown numerically and also graphically on a logarithmic scale. They are sorted in order of year of publication within study type (cohort, case-control). In the graphical representation individual RR estimates are indicated by a solid square, with the area of the square proportional to the weight (inverse-variance) of the estimate. Also shown is the combined estimate, derived by random-effects meta-analysis. This is represented by a diamond of standard height, with the width indicating the 95% CI. See Table 11 for further details relating to the estimates, and Table 12 for fuller details of the meta-analysis.

There are rather more data for the combined category of all cancers of the digestive system. Of the four studies providing data, all conducted in the USA, NHANES I [22] and CPS-II [23] show no relationship, CPS-I [23] a weak, but significant, positive relationship, and the case-control study of Sterling *et al*. [89] a significant negative relationship. Overall, the combined estimate (see Table 12 and Figure 6), all based on smoking-adjusted data, is 0.86 (0.59–1.25, *n *= 5), with significant evidence of heterogeneity (*P *= 0.002). The analysis for never smokers removes the case-control study and eliminates the heterogeneity. However the combined estimate of 1.14 (0.99–1.33, *n *= 4) remains non-significant.

**Table 12:** Overall digestive cancer; meta-analysis results

				Heterogeneity		
				
Type of ST (region)^a^	Adjustments/restrictions^b^	Number of estimates (RR/OR ids)^c^	Random-effects RR/OR (95% CI)	χ^2^	I^2^	*P*(χ^2^)
Any (USA)^d^	Overall data	5 (4, 5, 6, 9, 19)	0.86 (0.59–1.25)	17.3	76.9	0.002
	Smoking-adjusted	5 (4, 5, 6, 9, 19)	0.86 (0.59–1.25)	17.3	76.9	0.002
	Never smokers	4 (4, 5, 6, 9)	1.14 (0.99–1.33)	3.1	2.1	0.382

^a ^For each study/sex, the RR/OR for ST from Table 11 was included if available, otherwise that for chewing tobacco or snuff was used.

^b ^Smoking-adjusted includes estimates for smokers and non-smokers combined, adjusted for smoking if available, and estimates for never smokers otherwise.

^c ^The actual estimates included are identified by their RR/OR identification numbers as given in Table 11.

^d ^All the available data for overall digestive cancer are from US studies.

CI = confidence interval; ST = smokeless tobacco; OR = odds ratio; RR = relative risk.

More data are needed before any conclusion can be drawn for these cancers.

### Larynx and nasal cancer

**Figure 7:**
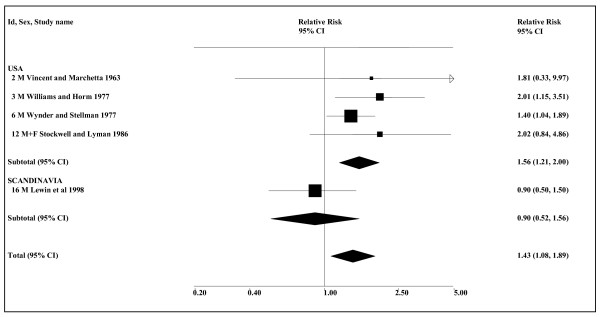
**Smokeless tobacco and larynx cancer by region (overall data)**. The five individual relative risk (RR) and 95% confidence interval (CI) estimates, separated by region, are shown numerically and also graphically on a logarithmic scale. They are sorted in order of year of publication within study type (cohort, case-control). In the graphical representation individual RR estimates are indicated by a solid square, with the area of the square proportional to the weight (inverse-variance) of the estimate. Also shown are the combined estimates, for the subgroups and overall, derived by random-effects meta-analysis. These are represented by a diamond of standard height, with the width indicating the 95% CI. See Table 13 for further details relating to the estimates, and Table 14 for fuller details of the meta-analyses. Only estimates 3 and 16 are smoking adjusted.

The data shown in Table 13 are quite limited. The evidence for nasal cancer is based on only three studies, none reporting a significant association with ST use. Seven studies investigated the relationship of ST to larynx cancer, two providing no effect estimates and merely reporting a lack of association. Control for confounding variables is very limited, with only two studies providing estimates adjusted for smoking, only one adjusting for alcohol and no study presenting any results for never smokers. The only study to adjust for smoking and alcohol [102], which shows no relationship of snuff to risk of larynx cancer, is the only study conducted in Scandinavia. Two US studies [55,56] report a significant relationship, however, and, as shown in Table 14 (see also Figure 7), an association is seen in the overall data (1.43, 1.08–1.89, *n *= 5).

**Table 13:** Larynx and nasal cancer; individual effect (relative risk/odds ratio) estimates

	ST use				RR/OR			
				
Source^a^	Type^b^	Exposure^c^	Smoking	Sex	Id.	Cases^d^	Estimate (95%CI)	Adjustment factors^e^
**Case-control studies**								
								
Wynder *et al*. 1957 [40]								
- larynx cancer	Chew	Ever	Any	M	1	NA	no association^f^	none
Vincent and Marchetta 1963 [45]								
- larynx cancer	Snuff	Use	Any	M	2	5	1.81 (0.33–9.97)	none
Williams and Horm 1977 [55]								
- larynx cancer	ST	Ever	Any	M	3	16	2.01 (1.15–3.51)^g^	age, race, smok
Wynder and Stellman 1977 [56]								
- larynx cancer	Chew	Ever	Any	M	4	46	1.35 (0.96–1.89)^g^	none
	Snuff				5	15	1.46 (0.82–2.57)^g^	none
	ST				6	61	1.40 (1.04–1.89)^h^	none
Engzell *et al*. 1978 [57]								
- nasal cancer	Snuff	Use	Any	M	7	NA	no association	none
Brinton *et al*. 1984 [64]								
- nasal cancer	Chew	Use	Any	M+F	8	15	0.74 (0.40–1.50)	sex
	Snuff				9	23	1.47 (0.80–2.80)	
	ST				10	38	1.08 (0.68–1.70)^h^	none
Stockwell and Lyman 1986 [70]								
- nasal cancer	ST	Ever	Any	M+F	11	1	2.93 (0.40–21.66)^g^	none
- larynx cancer	ST	Ever	Any	M+F	12	6	2.02 (0.84–4.86)^g^	none
Young *et al*. 1986 [71]								
- larynx cancer	ST	Ever	Any	M	13	NA	no association	none
Lewin *et al*. 1998 [102]								
- larynx cancer	Snuff	Current	Any	M	14	15	1.00 (0.50–1.90)	age, alc, res, smok
		Former			15	9	0.80 (0.40–1.70)	
		Ever			16	24	0.90 (0.50–1.50)	

^a ^Fuller details of the studies are given in Tables 1 and 2.

^b ^ST implies smokeless tobacco unspecified, or combined snuff use or chewing.

^c ^Ever, former and current ST use were compared with never ST. Use indicates timing not given and comparison is with non use.

^d ^'Id.' is the RR/OR identification number used in Table 14, and 'Cases' is the number of cases in ST users as defined. NA = not available.

^e ^Abbreviations used: alc = alcohol, res = area of residence, smok = smoking.

^f ^The average ridit duration of chewing was non-significantly lower in the larynx cancer cases.

^g ^RR/OR and/or 95% CI estimated from data provided in the source.

^h ^RR/OR and/or 95% CI estimated from data provided in the source assuming that no one both chewed and used snuff.

CI = confidence interval; ST = smokeless tobacco; OR = odds ratio; RR = relative risk.

**Table 14:** Larynx and nasal cancer; meta-analysis results

				Heterogeneity		
				
Type of ST (region)^a^	Adjustments/restrictions^b^	Number of estimates (RR/OR ids)^c^	Random-effects RR/OR (95% CI)	χ^2^	I^2^	*P*(χ^2^)
**Larynx cancer**^d^						
						
Any	Overall data	5 (2, 3, 6, 12, 16)	1.43 (1.08–1.89)	4.8	17.4	0.304
	Smoking-adjusted	2 (3, 16)	1.34 (0.61–2.95)	4.0	75.3	0.044
						
Any (USA)	Overall data	4 (2, 3, 6, 12)	1.56 (1.21–2.00)	1.7	0.0	0.646
	Smoking-adjusted	1 (3)	2.01 (1.15–3.51)	--	--	--
						
Snuff (Scandinavia)	Overall data	1 (16)	0.90 (0.50–1.50)	--	--	--
	Smoking-adjusted	1 (16)	0.90 (0.50–1.50)	--	--	--
						
**Nasal cancer**^e^						
Any	Overall data	2 (10, 11)	1.14 (0.73–1.77)	0.9	0.0	0.339

^a ^For each study/sex, the RR/OR for ST from Table 13 was included if available, otherwise that for chewing tobacco or snuff was used.

^b ^Smoking-adjusted includes estimates for smokers and non-smokers combined, adjusted for smoking if available, and estimates for never smokers otherwise.

^c ^The actual estimates included are identified by their RR/OR identification numbers as given in Table 13.

^d ^For larynx cancer there are no data for never smokers.

^e ^For nasal cancer the only data are from US studies and not smoking-adjusted.

CI = confidence interval; ST = smokeless tobacco; OR = odds ratio; RR = relative risk.

Given the independent role of smoking and alcohol in larynx cancer [7,8], and the lack of association in the one study that has adjusted for both these factors [102], any independent association of ST use with larynx cancer risk has not been established. More data are needed before any conclusion can be drawn on the role of ST in larynx and nasal cancers.

### Lung cancer

**Figure 8:**
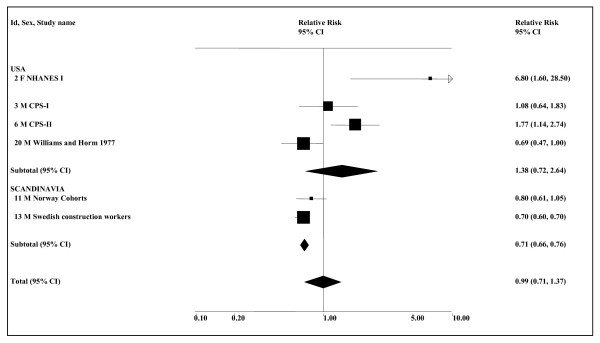
**Smokeless tobacco and lung cancer by region (smoking-adjusted data)**. The six individual smoking-adjusted relative risk (RR) and 95% confidence interval (CI) estimates, separated by region, are shown numerically and also graphically on a logarithmic scale. They are sorted in order of year of publication within study type (cohort, case-control). In the graphical representation individual RR estimates are indicated by a solid square, with the area of the square proportional to the weight (inverse-variance) of the estimate. Also shown are the combined estimates, for the subgroups and overall, derived by random-effects meta-analysis. These are represented by a diamond of standard height, with the width indicating the 95% CI. See Table 15 for further details relating to the estimates, and Table 16 for fuller details of the meta-analyses.

Table 15 summarises data from six cohort and three case-control studies. The case-control studies provide only estimates for smokers and non-smokers combined, and only one of these is adjusted for smoking. The cohort studies all provide estimates for never smokers, with two also giving smoking-adjusted results for the overall population. The meta-analyses (see Table 16 and Figure 8) show no evidence that ST use increases risk of lung cancer, with the combined estimate for smoking-adjusted data 0.99 (95% CI 0.71–1.37). However, there is considerable heterogeneity (*P *< 0.001), the major contributors to this being the high RR of 6.80 (1.60–28.5) in never smokers in NHANES I [22], the significant increase of 1.77 (1.14–2.74) from CPS-II [23], and the low RR of 0.70 (0.60–0.70) for the Swedish construction workers study [32]. While the combined estimate for never smokers for any ST use is greater than 1.0 (1.34, 0.80–2.23, *n *= 5), it is not statistically significant.

**Table 15:** Lung cancer; individual effect (relative risk/odds ratio) estimates

	ST use				RR/OR			
				
Source^a^	Type^b^	Exposure^c^	Smoking	Sex	Id.	Cases^d^	Estimate (95%CI)^e^	Adjustment factors^f^
**Cohort studies**								
								
US Veterans: Winn *et al*. 1982 [19]	ST	Ever	Never	M^g^	1	NA	0.60 (NA)	age
NHANES I: Accortt *et al*. 2005 [22]	ST	Ever	Never	F	2	4	6.80 (1.60–28.5)	age, pov, race
CPS-I: Henley *et al*. 2005 [23]	ST	Current	Never	M	3	18	1.08 (0.64–1.83)	age, alc, asp, bmi, diet, edu, exer, occ, race
CPS-II: Henley *et al*. 2005 [23]	ST	Current	Never	M	4	18	2.00 (1.23–3.24)	age, alc, asp, bmi, diet, edu, exer, occ, race
	ST	Former			5	4	1.17 (0.43–3.14)	
	ST	Ever			6	22	1.77 (1.14–2.74)^h^	
	Chew only	Current			7	12	1.97 (1.10–3.54)	
	Snuff only				8	2	2.08 (0.51–8.46)	
Norway cohorts: Boffetta *et al*. 2005 [26]	Snuff	Current	Any	M	9	44	0.80 (0.58–1.11)	age, smok
		Former			10	28	0.80 (0.54–1.19)	
		Ever			11	72	0.80 (0.61–1.05)	
		Ever	Never		12	3	0.96 (0.26–3.56)	age
Swedish construction workers: Luo *et al*. 2007 [32]	Snuff	Ever	Any	M	13	NA	0.70 (0.60–0.70)	age, bmi, smok
		Current	Never		14	15	0.80 (0.40–1.30)	age, bmi
		Former			15	3	0.90 (0.30–3.00)	
		Ever			16	18	0.80 (0.50–1.30)	
								
**Case-control studies**								
Doll and Hill 1952 [38]	Chew	Ever	Any	M	17	40	0.61 (0.41–0.92)^h^	none
	Snuff				18	33	0.76 (0.48–1.21)^h^	
	ST				19	73	0.66 (0.41–0.90)^h^	
Williams and Horm 1977 [55]	ST	Ever	Any	M	20	36	0.69 (0.47–1.00)^h^	age, race, smok
				F	21	1	0.38 (0.05–2.80)^h^	none
Wynder and Stellman 1977 [56]	Chew	Ever	Any	M	22	117	1.26 (0.99–1.59)^h^	none
	Snuff				23	35	1.25 (0.83–1.89)^h^	
	ST				24	152	1.27 (1.03–1.57)^h^	

^a ^Fuller details of the studies are given in Tables 1 and 2.

^b ^ST implies smokeless tobacco unspecified, or combined snuff use or chewing.

^c ^Ever, former and current ST use were compared with never ST. Use indicates timing not given and comparison is with non use.

^d ^'Id.' is the RR/OR identification number used in Table 16, and 'Cases' is the number of cases in ST users as defined. NA = not available.

^e ^NA = not available.

^f ^Abbreviations used: alc = alcohol consumption, asp = aspirin, bmi = body mass index, edu = education, exer = exercise, occ = occupation, pov = poverty, smok = smoking.

^g ^The population included < 0.5% females.

^h ^RR/OR and/or 95% CI estimated from data provided in the source.

CI = confidence interval; ST = smokeless tobacco; OR = odds ratio; RR = relative risk.

**Table 16:** Lung cancer; meta-analysis results

				Heterogeneity		
				
Type of ST (region)^a^	Adjustments/restrictions^b^	Number of estimates (RR/OR ids)^c^	Random-effects RR/OR (95% CI)	χ^2^	I^2^	*P*(χ^2^)
Any	Overall data	9 (2, 3, 6, 11, 13, 19, 20, 21, 24)	0.96 (0.73–1.27)	53.2	85.0	< 0.001
	Smoking-adjusted	6 (2, 3, 6, 11, 13, 20)	0.99 (0.71–1.37)^d^	28.7	82.6	< 0.001
	Never smokers	5 (2, 3, 6, 12, 16)	1.34 (0.80–2.23)	11.5	65.3	0.021
						
Any (USA)	Overall data	6 (2, 3, 6, 20, 21, 24)	1.22 (0.82–1.83)	18.5	73.0	0.002
	Smoking-adjusted	4 (2, 3, 6, 20)	1.38 (0.72–2.64)	16.5	81.9	0.001
	Never smokers	3 (2, 3, 6)	1.79 (0.91–3.51)	6.2	67.8	0.045
						
Snuff (Scandinavia)	Overall data	2 (11, 13)	0.71 (0.66–0.76)	0.9	0.0	0.354
	Smoking-adjusted	2 (11, 13)	0.71 (0.66–0.76)	0.9	0.0	0.354
	Never smokers	2 (12, 16)	0.82 (0.52–1.28)	0.1	0.0	0.798

^a ^For each study/sex, the RR/OR for ST from Table 15 was included if available, otherwise that for chewing tobacco or snuff was used.

^b ^Smoking-adjusted includes estimates for smokers and non-smokers combined, adjusted for smoking if available, and estimates for never smokers otherwise.

^c ^The actual estimates included are identified by their RR/OR identification numbers as given in Table 15.

^d ^Test for publication bias 0.05 ≤ *P *< 0.1.

CI = confidence interval; ST = smokeless tobacco; OR = odds ratio; RR = relative risk.

While the data have unexplained heterogeneity, they do not provide any clear indication of a relationship of lung cancer to ST use.

Not included in Table 15 are results from an analysis conducted by Henley *et al*. in 2007 [25] based on follow-up of the CPS-II cohort from 1982 to 2002. They report an increased risk of lung cancer (1.46, 1.24–1.73) in men who switched from cigarette smoking to ST compared with those who quit entirely, after adjusting for age, other demographic variables, as well as variables associated with smoking history. This analysis may be biased by reliance on tobacco use data recorded in 1982, and by residual confounding, with the paper reporting marked differences between switchers and quitters in a range of characteristics, with adjustment substantially reducing the RR estimate from the age-adjusted estimate of 1.92 (1.63–3.26).

### Prostate cancer

**Figure 9:**
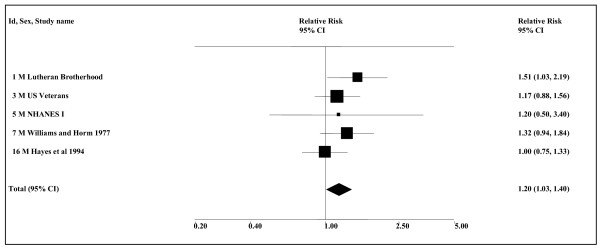
**Smokeless tobacco and prostate cancer (USA overall data)**. The five individual relative risk (RR) and 95% confidence interval (CI) estimates, all for the USA, are shown numerically and also graphically on a logarithmic scale. They are sorted in order of year of publication within study type (cohort, case-control). In the graphical representation individual RR estimates are indicated by a solid square, with the area of the square proportional to the weight (inverse-variance) of the estimate. Also shown are the combined estimates, for the subgroups and overall, derived by random-effects meta-analysis. These are represented by a diamond of standard height, with the width indicating the 95% CI. See Table 17 for further details relating to the estimates, and Table 18 for fuller details of the meta-analyses.

Table 17 presents data from five cohort and two case-control studies, all conducted in the USA. No significant association between ST and prostate cancer is evident in five studies, but significant increases are seen in the Lutheran Brotherhood Study [11] and, for current snuff users only, in the case-control study by Hayes *et al*. [96]. Based on the five studies which provide usable data, the overall estimate (see Table 18 and Figure 9) is 1.20 (95% CI 1.03–1.40).

**Table 17:** Prostate cancer; individual effect (relative risk/odds ratio) estimates

	ST use			RR/OR			
			
Source^a^	Type^b^	Exposure^c^	Smoking	Id.	Cases^d^	Estimate (95%CI)	Adjustment factors^e^
**Cohort studies**							
							
Lutheran Brotherhood: Hsing *et al*. 1990 [11]	ST	Ever	Any	1	38	1.51 (1.03–2.19)^f^	age, smok
			Never	2	10	4.50 (2.10–9.70)	age
US Veterans: Hsing *et al*. 1991 [15]	ST	Ever	Never	3	48	1.17 (0.88–1.56)	age
Iowa cohort: Putnam *et al*. 2000 [20]	ST	Ever	Any	4	NA	no association	age
NHANES I: Accortt *et al*. 2005 [22]	ST	Ever	Never	5	19	1.20 (0.50–3.40)	age, pov, race
Norway cohorts: IARC Monograph 37 1985 [14]	ST	Use	Any	6	NA	no association	age, res, smok
							
**Case-control studies**							
Williams and Horm 1977 [55]	ST	Ever	Any	7	65	1.32 (0.94–1.84)^f^	age, race, smok
Hayes *et al*. 1994 [96]	Chew	Current	Any	8	14	0.56 (0.30–1.06)^f^	none
		Former		9	56	1.08 (0.75–1.55)^f^	
		Ever		10	70	0.91 (0.67–1.25)^f^	
	Snuff	Current	Any	11	10	6.74 (1.47–30.84)^f^	
		Former		12	10	0.79 (0.36–1.74)^f^	
		Ever		13	20	1.42 (0.75–2.67)^f^	
	ST	Current	Any	14	24	0.92 (0.54–1.58)^g^	
		Former		15	66	1.03 (0.74–1.43)^g^	
		Ever		16	90	1.00 (0.75–1.33)^g^	

^a ^Fuller details of the studies are given in Tables 1 and 2.

^b ^ST implies smokeless tobacco unspecified, or combined snuff use or chewing.

^c ^Ever, former and current ST use were compared with never ST. Use indicates timing not given and comparison is with non use.

^d ^'Id.' is the RR/OR identification number used in Table 18, and 'Cases' is the number of cases in ST users as defined. NA = not available.

^e ^Abbreviations used: pov = poverty, res = area of residence, smok = smoking.

^f ^RR/OR and/or 95% CI estimated from data provided in the source.

^g ^RR/OR and/or 95% CI estimated from data provided in the source assuming that no one both chewed and used snuff.

CI = confidence interval; ST = smokeless tobacco; OR = odds ratio; RR = relative risk.

**Table 18:** Prostate cancer; meta-analysis results

				Heterogeneity		
				
Type of ST (region)^a^	Adjustments/restrictions^b^	Number of estimates (RR/OR ids)^c^	Random-effects RR/OR (95% CI)	χ^2^	I^2^	*P*(χ^2^)
Any^d^	Overall data	5 (1, 3, 5, 7, 16)	1.20 (1.03–1.40)	3.3	0.0	0.506
	Smoking-adjusted	4 (1, 3, 5, 7)	1.29 (1.07–1.55)	1.2	0.0	0.764
	Never smokers	3 (2, 3, 5)	1.81 (0.76–4.30)	10.5	81.0	0.005

^a ^For each study/sex, the RR/OR for ST from Table 17 was included if available, otherwise that for chewing tobacco or snuff was used.

^b ^Smoking-adjusted includes estimates for smokers and non-smokers combined, adjusted for smoking if available, and estimates for never smokers otherwise.

^c ^The actual estimates included are identified by their RR/OR identification numbers as given in Table 17.

^d ^All the available data for prostate cancer are from US studies.

CI = confidence interval; ST = smokeless tobacco; OR = odds ratio; RR = relative risk.

Prostate cancer is not considered smoking related [7,8], and more information on its relationship with ST is needed before any clear conclusion can be drawn.

### Bladder cancer

**Figure 10:**
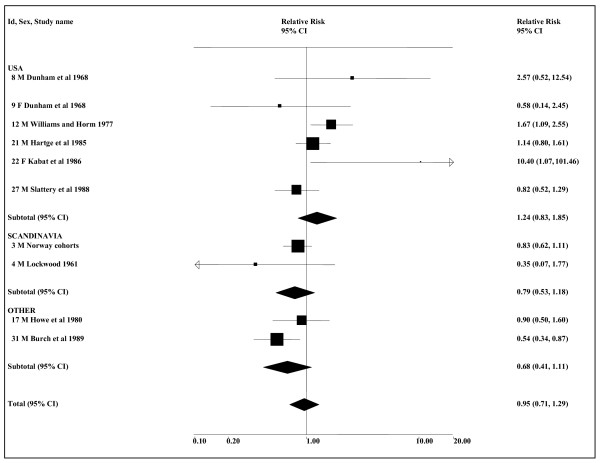
**Smokeless tobacco and bladder cancer by region (smoking-adjusted data)**. The 10 individual smoking-adjusted relative risk (RR) and 95% confidence interval (CI) estimates, separated by region, are shown numerically and also graphically on a logarithmic scale. They are sorted in order of year of publication within study type (cohort, case-control). In the graphical representation individual RR estimates are indicated by a solid square, with the area of the square proportional to the weight (inverse-variance) of the estimate. Also shown are the combined estimates, for the subgroups and overall, derived by random-effects meta-analysis. These are represented by a diamond of standard height, with the width indicating the 95% CI. See Table 19 for further details relating to the estimates, and Table 20 for fuller details of the meta-analyses.

Table 19 summarises data from the Norway cohorts study [26] and from 12 case-control studies. None of the case-control studies were conducted after 1990, and with the exception of two studies in Denmark [43,62], all were carried out in the USA or Canada. The great majority of the estimates are non-significant, and based on 10 smoking-adjusted estimates the overall estimate (see Table 20 and Figure 10) is 0.95 (95% CI 0.71–1.29). However, there is significant heterogeneity due mainly to estimates 8, 12 and 22, which show a positive association, the last two of which are significant, and estimate 31 which shows a significant negative association.

**Table 19:** Bladder cancer; individual effect (relative risk/odds ratio) estimates

	ST use				RR/OR			
				
Source^a^	Type^b^	Exposure^c^	Smoking	Sex	Id.	Cases^d^	Estimate (95%CI)	Adjustment factors^e^
**Cohort studies**								
Norway cohorts: Boffetta *et al*. 2005 [26]	Snuff	Current	Any	M	1	40	0.72 (0.52–1.06)	age, smok
		Former			2	30	0.98 (0.66–1.47)	
		Ever			3	69	0.83 (0.62–1.11)	
								
**Case-control studies**								
Lockwood 1961 [43]	ST	Current	Never	M	4	2	0.35 (0.07–1.77)^f^	none
								
Wynder *et al*. 1963 [46]	Chew	Ever	Any	M	5	33	1.42 (0.82–2.47)^f^	none
	Snuff				6	6	0.66 (0.23–1.88)^f^	
	ST				7	39	1.21 (0.74–1.98)^g^	
								
Dunham *et al*. 1968 [48]	ST	Ever	Never	M	8	4	2.57 (0.52–12.54)^f^	race
				F	9	3	0.58 (0.14–2.45)^f^	
								
Cole *et al*. 1971 [51]	Chew	Ever	Any	M	10	46	no association^h^	age
	Snuff				11	3	no association^i^	
								
Williams and Horm 1977 [55]	ST	Ever	Any	M	12	29	1.67 (1.09–2.55)^f^	age, race, smok
				F	13	1	0.82 (0.11–6.02)^f^	none
								
Wynder and Stellman 1977 [56]	Chew	Ever	Any	M	14	47	0.87 (0.63–1.21)^f^	none
	Snuff				15	11	0.69 (0.36–1.31)^f^	
	ST				16	58	0.82 (0.61–1.10)^g^	
								
Howe *et al*. 1980 [58]	Chew	Ever	Any	M	17	NA	0.90 (0.50–1.60)	age, smok
								
Mommsen and Aagaard 1983 [62]	Chew	Ever	Any	M	18	39	1.70 (1.00–2.90)	age, res
								
Hartge *et al*. 1985 [66]	Chew	Ever	Never^j^	M	19	40	1.02 (0.67–1.54)	age, race, res, smok^j^
	Snuff				20	11	0.77 (0.38–1.56)	
	ST				21	51	1.14 (0.80–1.61)^g^	none
								
Kabat *et al*. 1986 [69]	Snuff	Ever	Never	F	22	3	10.40 (1.07–101.46)	none
								
Slattery *et al*. 1988 [77]	Chew	Ever	Any	M	23	20	0.76 (0.42–1.39)	smok^k^
			Never		24	1	0.36 (0.05–2.82)^l^	none
	Snuff	Ever	Any		25	16	0.92 (0.47–1.82)	smok^k^
			Never		26	2	2.74 (0.45–16.69)^m^	none
	ST	Ever	Any		27	36	0.82 (0.52–1.29)^g^	smok^k^
			Never		28	3	0.86 (0.24–3.07)^g^	none
								
Burch *et al*. 1989 [79]	Chew	Ever	Any	M	29	26	0.60 (0.34–1.06)	age, res, smok
	Snuff				30	9	0.47 (0.21–1.07)	
	ST				31	35	0.54 (0.34–0.87)^g^	

^a ^Fuller details of the studies are given in Tables 1 and 2.

^b ^ST implies smokeless tobacco unspecified, or combined snuff use or chewing.

^c ^Ever, former and current ST use were compared with never ST. Use indicates timing not given and comparison is with non use.

^d ^'Id.' is the RR/OR identification number used in Table 20, and 'Cases' is the number of cases in ST users as defined. NA = not available.

^e ^Abbreviations used: res = area of residence, smok = smoking.

^f ^RR/OR and/or 95% CI estimated from data provided in the source.

^g ^RR/OR and/or 95% CI estimated from data provided in the source assuming that no one both chewed and used snuff.

^h ^Age-adjusted expected number of cases who chewed tobacco was given as 42.3 versus 46 observed.

^i ^Age-adjusted expected number of cases who used snuff was given as 2.9 versus 3 observed.

^j ^Estimates were for never cigarette smokers adjusted for other tobacco use.

^k ^Adjusted for age started to smoke; results adjusted for smoking group, pack years or years stopped are similar.

^l ^The source paper gave 2.78 (0.38–20.20) which is incorrect based on the numbers in the 2 × 2 table.

^m ^The source paper gave 2.73 (0.48–15.57) which is incorrect based on the numbers in the 2 × 2 table.

CI = confidence interval; ST = smokeless tobacco; OR = odds ratio; RR = relative risk.

**Table 20:** Bladder cancer; meta-analysis results

				Heterogeneity		
				
Type of ST (region)^a^	Adjustments/restrictions^b^	Number of estimates (RR/OR ids)^c^	Random-effects RR/OR (95% CI)	χ^2^	I^2^	*P*(χ^2^)
Any	Overall data	14 (3, 4, 7, 8, 9, 12, 13, 16, 17, 18, 21, 22, 27, 31)	1.00 (0.80–1.25)	28.7	54.7	0.007
	Smoking-adjusted	10 (3, 4, 8, 9, 12, 17, 21, 22, 27, 31)	0.95 (0.71–1.29)	22.3	59.6	0.008
	Never smokers	6 (4, 8, 9, 21, 22, 28)	1.10 (0.60–2.02)	7.7	35.1	0.173
						
Any (USA)	Overall data	9 (7, 8, 9, 12, 13, 16, 21, 22, 27)	1.11 (0.85–1.45)	14.8	45.9	0.064
	Smoking-adjusted	6 (8, 9, 12, 21, 22, 27)	1.24 (0.83–1.85)	10.4	52.1	0.064
	Never smokers	5 (8, 9, 21, 22, 28)	1.25 (0.69–2.26)	5.6	29.2	0.227
						
Snuff (Scandinavia)^d^	Overall data	1 (3)	0.83 (0.62–1.11)	--	--	--
	Smoking-adjusted	1 (3)	0.83 (0.62–1.11)	--	--	--

^a ^For each study/sex, the RR/OR for ST from Table 19 was included if available, otherwise that for chewing tobacco or snuff was used.

^b ^Smoking-adjusted includes estimates for smokers and non-smokers combined, adjusted for smoking if available, and estimates for never smokers otherwise.

^c ^The actual estimates included are identified by their RR/OR identification numbers as given in Table 19.

^d ^There are no data for never smokers for snuff in Scandinavia.

CI = confidence interval; ST = smokeless tobacco; OR = odds ratio; RR = relative risk.

Considered together, the data provide no real evidence of an association between ST and bladder cancer.

### Kidney cancer

**Figure 11:**
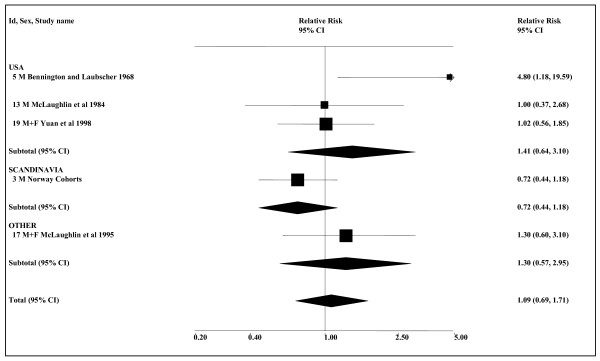
**Smokeless tobacco and kidney cancer by region (smoking-adjusted data)**. The five individual smoking-adjusted relative risk (RR) and 95% confidence interval (CI) estimates, separated by region, are shown numerically and also graphically on a logarithmic scale. They are sorted in order of year of publication within study type (cohort, case-control). In the graphical representation individual RR estimates are indicated by a solid square, with the area of the square proportional to the weight (inverse-variance) of the estimate. Also shown are the combined estimates, for the subgroups and overall, derived by random-effects meta-analysis. These are represented by a diamond of standard height, with the width indicating the 95% CI. See Table 21 for further details relating to the estimates, and Table 22 for fuller details of the meta-analyses.

Table 21 summarises evidence from one cohort and nine case-control studies, none conducted in Sweden. The estimates are generally based on small numbers of cases using ST, and are variable, with four studies [47,68,73,100] providing a statistically significant OR estimate exceeding 3.0, and other studies (and other estimates from the four studies) showing notably smaller estimates, that are not significant. Most of the meta-analysis estimates shown in Table 22 (see also Figure 11) are elevated, with some evidence of heterogeneity, but none are statistically significant. Based on five smoking-adjusted estimates the overall estimate for any ST use is 1.09 (0.69–1.71).

**Table 21:** Kidney cancer; individual effect (relative risk/odds ratio) estimates

	ST use				RR/OR			
				
Source^a^	Type^b^	Exposure^c^	Smoking	Sex	Id.	Cases^d^	Estimate (95%CI)	Adjustment factors^e^
**Cohort studies**								
								
Norway cohorts: Boffetta *et al*. 2005 [26]	Snuff	Current	Any	M	1	9	0.47 (0.23–0.94)	age, smok
		Former			2	13	1.17 (0.63–2.16)	
		Ever			3	22	0.72 (0.44–1.18)	
								
**Case-control studies**								
Bennington and Laubscher 1968 [47]	Chew	Use	Any	M	4	5	1.22 (0.39–3.85)^f^	none
			Never		5	5	4.80 (1.18–19.59)^f^	age
Armstrong *et al*. 1976 [53]	ST	Current	Any	M	6	6	0.98 (0.30–3.15)^f^	none
		Former			7	6	0.73 (0.24–2.20)^f^	
		Ever			8	12	0.84 (0.37–1.92)^f^	
Williams and Horm 1977 [55]	ST	Ever	Any	M	9	3	0.59 (0.18–1.90)^f^	none
				F	10	1	1.26 (0.17–9.33)^f^	
McLaughlin *et al*. 1984 [65]	Chew	Use	Any	M	11	NA	0.40 (0.10–2.60)	age, smok
	Snuff				12	NA	1.70 (0.50–6.00)	
	ST				13	NA	1.00 (0.37–2.68)^g^	
Goodman *et al*. 1986 [68]	Chew	Ever	Any	M	14^h^	13	4.00 (1.13 – 14.17)	age, hosp, race, tadm
Asal *et al*. 1988 [73]	Snuff	Use	Any	M	15^i^	NA	3.60 (1.20–13.30)	age, hosp, race, tadm
					16^j^	NA	no association	age, race, tadm
McLaughlin *et al*. 1995 [99]	ST	Use	Never	M+F	17	11	1.30 (0.60–3.10)	age, bmi, res, sex
Muscat *et al*. 1995 [100]	Chew	Ever	Any	M	18	14	3.20 (1.10–8.70)	age, edu
Yuan *et al*. 1998 [106]	ST	Ever	Any	M+F	19	32	1.02 (0.56–1.85)	age, edu, smok

^a ^Fuller details of the studies are given in Tables 1 and 2.

^b ^ST implies smokeless tobacco unspecified, or combined snuff use or chewing.

^c ^Ever, former and current ST use were compared with never ST. Use indicates timing not given and comparison is with non use.

^d ^'Id.' is the RR/OR identification number used in Table 22, and 'Cases' is the number of cases in ST users as defined. NA = not available.

^e ^Abbreviations used: bmi = body mass index, edu = education, hosp = hospital, res = residence, smok = smoking, tadm = time of admission.

^f ^RR/OR and/or 95% CI estimated from data provided in the source.

^g ^Estimated assuming ORs for chewing and snuff are independent.

^h ^The authors also report the results of an analysis adjusting for the effects of the matching factors, body mass index, decaffeinated coffee use and continuous pack-years of cigarette smoking. The authors estimated an OR (95% CI) of 0.87 (0.15–5.14) for the effect of chewing among never smokers of cigarettes, and of 26.00 (4.41–153.00) for the joint effect of pack-years cigarette smoking and chewing tobacco use. These results could not readily be incorporated into the meta-analyses as no overall estimate for chewing tobacco use adjusted for cigarette smoking was available.

^i ^Analysis uses hospital controls.

^j ^Analysis uses population controls.

CI = confidence interval; ST = smokeless tobacco; OR = odds ratio; RR = relative risk.

**Table 22:** Kidney cancer; meta-analysis results

				Heterogeneity		
				
Type of ST (region)^a^	Adjustments/restrictions^b^	Number of estimates (RR/OR ids)^c^	Random-effects RR/OR (95% CI)	χ^2^	I^2^	*P*(χ^2^)
Any	Overall data	11 (3, 4, 8, 9, 10, 13, 14, 15, 17, 18, 19)	1.23 (0.86–1.76)^d^	16.5	39.2	0.087
	Smoking-adjusted	5 (3, 5, 13, 17, 19)	1.09 (0.69–1.71)^e^	6.9	41.9	0.142
	Never smokers	2 (5, 17)	2.19 (0.63–7.70)	2.5	59.6	0.116
						
Any (USA)	Overall data	8 (4, 9, 10, 13, 14, 15, 18, 19)	1.52 (0.94–2.46)	11.1	37.1	0.133
	Smoking-adjusted	3 (5, 13, 19)	1.41 (0.64–3.10)	4.2	51.8	0.125
	Never smokers	1 (5)	4.80 (1.18–19.56)	--	--	--
						
Snuff (Scandinavia)^f^	Overall data	1 (3)	0.72 (0.44–1.18)	--	--	--
	Smoking-adjusted	1 (3)	0.72 (0.44–1.18)	--	--	--

^a ^For each study/sex, the RR/OR for ST from Table 21 was included if available, otherwise that for chewing tobacco or snuff was used.

^b ^Smoking-adjusted includes estimates for smokers and non-smokers combined, adjusted for smoking if available, and estimates for never smokers otherwise.

^c ^The actual estimates included are identified by their RR/OR identification numbers as given in Table 21.

^d ^Test for publication bias 0.05 ≤ *P *< 0.1.

^e ^Test for publication bias 0.01 ≤ *P *< 0.05.

^f ^There are no available data for never smokers using snuff in Scandinavia.

CI = confidence interval; ST = smokeless tobacco; OR = odds ratio; RR = relative risk.

While there is a suggestion of a possible relationship, more data are needed before any firm conclusions can be reached.

### Haematopoietic and lymphoid cancer

**Figure 12:**
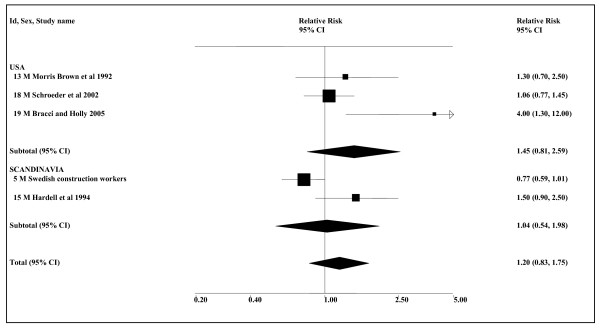
**Smokeless tobacco and non-Hodgkin's lymphoma by region (overall data)**. The five individual relative risk (RR) and 95% confidence interval (CI) estimates, separated by region, are shown numerically and also graphically on a logarithmic scale. They are sorted in order of year of publication within study type (cohort, case-control). In the graphical representation individual RR estimates are indicated by a solid square, with the area of the square proportional to the weight (inverse-variance) of the estimate. Also shown are the combined estimates, for the subgroups and overall, derived by random-effects meta-analysis. These are represented by a diamond of standard height, with the width indicating the 95% CI. See Table 23 for further details relating to the estimates, and Table 24 for fuller details of the meta-analyses. Only estimates 5, 13 and 19 are smoking-adjusted.

Table 23 summarises evidence from three cohort and seven case-control studies for overall haematopoietic cancer and for specific types. The only report of a significant association is the OR of 4.0 (1.3–12.0) for non-Hodgkin's lymphoma in the case-control study of Bracci and Holly [112]. However, the combined evidence from the five studies (see Table 24 and Figure 12) for non-Hodgkin's lymphoma shows no significant relationship (1.20, 0.83–1.75), though there is significant heterogeneity (*P *= 0.01), due mainly to the Bracci and Holly estimate. The evidence for other endpoints – multiple myeloma, Hodgkin's disease, leukaemia, and overall haematopoietic cancer – is more limited, and does not suggest any relationship with ST use.

**Table 23:** Haematopoietic and lymphoid cancer; individual effect (relative risk/odds ratio) estimates

	ST use				RR/OR			
				
Source^a^	Type^b^	Exposure^c^	Smoking	Sex	Id.	Cases^d^	Estimate (95%CI)	Adjustment factors^e^
**Cohort studies**								
								
US veterans: Heinemann *et al*. 1992 [16]								
- multiple myeloma	ST	Use	Never	M^f^	1	6	1.00 (0.40–2.30)	age, time, yriv
CPS-II: Henley *et al*. 2005 [23]								
- any haematopoietic cancer	ST	Current	Never	M	2	19	0.95 (0.60–1.51)	age, alc, asp, bmi, diet, edu, exer, occ, race
		Former			3	9	1.16 (0.60–2.25)	
		Ever			4	28	1.01 (0.69–1.48)^g^	
Swedish construction workers: Fernberg *et al*. 2006 [30]								
- non-Hodgkin's lymphoma	Snuff	Ever	Never	M	5	66	0.77 (0.59–1.01)	age, bmi
- Hodgkin's disease	Snuff	Ever	Never	M	6	15	0.88 (0.49–1.58)	
Swedish construction workers: Fernberg *et al*. 2007 [31]								
- leukaemia	Snuff	Ever	Never	M	7	NA	no increased risk	age, bmi
- multiple myeloma	Snuff	Ever	Never	M	8	NA	no increased risk	age, bmi
								
**Case-control studies**								
Williams and Horm 1977 [55]								
- any haemopoietic cancer	ST	Ever	Any	M	9	13	0.63 (0.35–1.14)^g^	none
				F	10	3	1.01 (0.31–3.29)^g^	
Lindquist *et al*. 1987 [72]								
- leukaemia	Snuff	Ever	Any	M+F	11	18	0.94 (0.47–1.89)^h^	age, res, sex
Morris Brown *et al*. 1992 [87]								
- leukaemia	ST	Use	Never	M	12	24	1.80 (0.90–3.30)^i^	age, alc, res
Morris Brown *et al*. 1992 [88]								
- non-Hodgkin's lymphoma	ST	Use	Never	M	13	19	1.30 (0.70–2.50)^j^	age, res
- multiple myeloma	ST	Use	Never	M	14	5	1.90 (0.50–6.60)	age, res
Hardell *et al*. 1994 [95]								
- non-Hodgkin's lymphoma	Snuff	Use	Any	M	15	35	1.50 (0.90–2.50)	none
Schroeder *et al*. 2002 [110]								
- non-Hodgkin's lymphoma	Chew	Ever	Any	M	16	19	1.23 (0.80–1.88)^k^	age, res
	Snuff				17	19	0.93 (0.61–1.41)^k^	
	ST				18	38	1.06 (0.77–1.45)^l^	
Bracci and Holly 2005 [112]								
- non-Hodgkin's lymphoma	ST	Ever	Never	M	19	7	4.00 (1.30–12.00)	age, alc, edu

^a ^Fuller details of the studies are given in Tables 1 and 2.

^b ^ST implies smokeless tobacco unspecified, or combined snuff use or chewing.

^c ^Ever, former and current ST use were compared with never ST. Use indicates timing not given and comparison is with non use.

^d ^'Id.' is the RR/OR identification number used in Table 24, and 'Cases' is the number of cases in ST users as defined. NA = not available.

^e ^Abbreviations used: alc = alcohol, asp = aspirin, bmi = body mass index, edu = education, exer = exercise, occ = occupation, smok = smoking, tadm = time of admission, yriv = year of interview.

^f ^The population included < 0.5% females.

^g ^Estimated from data on limited number of exposed cases for eight sub-types of haemopoietic cancer.

^h ^RR/OR and/or 95% CI estimated from data provided in the source.

^i ^Data for six subtypes of leukaemia were also provided, but none were statistically significant.

^j ^Data for five subtypes of non-Hodgkin's lymphoma were also provided, but none were statistically significant.

^k ^Estimated from data for t (14,18)-positive and t (14,18)-negative cases.

^l ^Estimated from the results for chew and snuff, assuming that no one both chewed and used snuff.

CI = confidence interval; ST = smokeless tobacco; OR = odds ratio; RR = relative risk.

**Table 24:** Non-Hodgkin's lymphoma; meta-analysis results

				Heterogeneity		
				
Type of ST (region)^a^	Adjustments/restrictions^b^	Number of estimates (RR/OR ids)^c^	Random-effects RR/OR (95% CI)	χ^2^	I^2^	*P*(χ^2^)
Any	Overall data	5 (5, 13, 15, 18, 19)	1.20 (0.83–1.75)^d^	12.8	68.8	0.012
	Smoking-adjusted	3 (5, 13, 19)	1.35 (0.62–2.94)	9.5	78.9	0.009
	Never smokers	3 (5, 13, 19)	1.35 (0.62–2.94)	9.5	78.9	0.009
						
Any (USA)	Overall data	3 (13, 18, 19)	1.45 (0.81–2.59)	5.2	61.2	0.076
	Smoking-adjusted	2 (13, 19)	2.07 (0.70–6.13)	3.0	66.2	0.085
	Never smokers	2 (13, 19)	2.07 (0.70–6.13)	3.0	66.2	0.085
						
Snuff (Scandinavia)	Overall data	2 (5, 15)	1.04 (0.54–1.98)	5.1	80.5	0.024
	Smoking-adjusted	1 (5)	0.77 (0.59–1.01)	--	--	--
	Never smokers	1 (5)	0.77 (0.59–1.01)	--	--	--

^a ^For each study/sex, the RR/OR for ST from Table 23 was included if available, otherwise that for chewing tobacco or snuff was used.

^b ^Smoking-adjusted includes estimates for smokers and non-smokers combined, adjusted for smoking if available, and estimates for never smokers otherwise.

^c ^The actual estimates included are identified by their RR/OR identification numbers as given in Table 23.

^d ^Test for publication bias 0.01 ≤ *P *< 0.05.

CI = confidence interval; ST = smokeless tobacco; OR = odds ratio; RR = relative risk.

### Other cancers

Table 25 summarises evidence from six cohort and four case-control studies relating to cancers of types not considered in Tables 3 to 24. Most of the results relate to specific cancer types, though some relate to broader groupings, such as genitourinary cancer and smoking-related cancer, which include cancer types considered earlier. Due to the variety of types, and the limited numbers of estimates relating to any one type, no meta-analyses were attempted. One of the studies [109] simply reported a lack of association (with glioma), and the remaining studies provided a total of 24 effect estimates with CI. Six of these are statistically significant. Zahm *et al*. [81] report an age-adjusted OR of 1.80 (95% CI 1.10–2.90) for soft tissue sarcoma based on a case-control study, though fail to confirm this later using data from the US Veterans Study [17]. The Williams and Horm study [55] provides a smoking-adjusted estimate of 4.18 (2.08–8.43) for cancer of the cervix, no other study giving relevant results. Moore *et al*. [39], in a study conducted in 1953, report a crude estimate of 2.41 (1.09–5.35) for cancer of the face, again an endpoint not considered by others. Roosaar *et al*. [35] report an increased risk of smoking-related cancer (1.6, 1.1–2.5) for never smokers, but not in a smoking-adjusted analysis for smoker and non-smokers combined (1.1, 0.8–1.4). Finally, based on the Swedish construction workers study, Odenbro *et al*. [29,33] report that snuff use is associated with a reduced smoking-adjusted risk of cutaneous squamous cell carcinoma (0.64, 0.44–0.95) and, in never smokers, with a reduced risk of melanoma (0.65, 0.52–0.82). These isolated reports need confirmation in other studies before any effect of ST can reliably be inferred. A study in Cherokee women [125,126] which shows no association of breast cancer with ever ST use, with an odds ratio adjusted for age at diagnosis estimated as 1.24 (0.26–6.02), is not considered in Table 25 as the study is of cross-sectional design. It contributes little to the evidence.

**Table 25:** Other cancers; individual effect (relative risk/odds ratio) estimates

	ST use				RR/OR			
				
Source^a^	Type^b^	Exposure^c^	Smoking	Sex	Id.	Cases^d^	Estimate (95%CI)	Adjustment factors^e^
**Cohort studies**								
								
US Veterans: Zahm *et al*. 1992 [17]								
- soft tissue sarcoma	ST	Ever	Any	M^f^	1	21	0.85 (0.53–1.36)	age, smok, time
NHANES I: Accortt *et al*. 2005 [22]								
- breast cancer	ST	Ever	Never	F	2	5	1.80 (0.50–6.50)	age, pov, race
CPS-I: Henley *et al*. 2005 [23]								
- genitourinary cancer	ST	Current	Never	M	3	98	0.97 (0.77–1.22)	age, alc, asp, bmi, diet, edu, exer, occ, race
CPS-II: Henley *et al*. 2005 [23]								
- genitourinary cancer	ST	Current	Never	M	4	44	1.15 (0.85–1.56)	age, alc, asp, bmi, diet, edu, exer, occ, race
		Former			5	16	0.97 (0.59–1.59)	
		Ever			6	60	1.10 (0.84–1.42)^g^	
Swedish construction workers: Odenbro *et al*. 2005 [29]								
- cutaneous squamous cell carcinoma	Snuff	Ever	Any	M	7	29	0.64 (0.44–0.95)	age, smok
Swedish construction workers: Odenbro *et al*. 2007 [33]								
- melanoma^h^	Snuff	Ever	Never	M	8	96	0.65 (0.52–0.82)	age, bir, bmi
Uppsala County: Roosaar *et al*. 2008 [35]								
- smoking related cancer	Snuff	Ever	Any	M	9	71	1.10 (0.80–1.40)	age, alc, res, smok, time
			Never		10	39	1.60 (1.10–2.50)	age, alc, res, time
								
**Case-control studies**								
								
Moore *et al*. 1953 [39]								
- cancer of face	ST	Use	Any	M	11	49	2.41 (1.09–5.35)	none
Williams and Horm 1977 [55]								
- breast cancer	ST	Ever	Any	F	12	11	0.60 (0.31–1.17)^g^	age, smok
- cancer of male genitalia	ST	Ever	Any	M	13	2	0.47 (0.11–1.94)^g^	None
- cancer of cervix	ST	Ever	Any	F	14	10	4.18 (2.08–8.43)^g^	age, smok
- cancer of uterus	ST	Ever	Any	F	15	7	1.92 (0.86–4.28)^g^	age, smok
- cancer of ovary	ST	Ever	Any	F	16	2	0.77 (0.19–3.21)^g^	none
- cancer of vulva	ST	Ever	Any	F	17	1	2.06 (0.28–15.41)^g^	none
- connective tissue	ST	Ever	Any	M	18	1	0.26 (0.04–1.93)^g^	none
- melanoma	ST	Ever	Any	M	19	1	0.30 (0.04–2.18)^g^	none
- nervous system cancer	ST	Ever	Any	M	20	1	0.18 (0.02–1.32)^g^	none
				F	21	2	3.28 (0.77–13.99)^g^	
- thyroid cancer	ST	Ever	Any	M	22	1	0.36 (0.05–2.69)^g^	none
				F	23	1	0.73 (0.10–5.38)^g^	
Zahm *et al*. 1989 [81]								
- soft tissue sarcoma	ST	Ever	Any	M	24	28	1.80 (1.10–2.90)	Age
Zheng *et al*. 2001 [109]								
- brain cancer (glioma)	Chew	Use	Any	M+F	25	NA	no association	NA
	Snuff				26	NA	no association	

^a ^Fuller details of the studies are given in Tables 1 and 2.

^b ^ST implies smokeless tobacco unspecified, or combined snuff use or chewing.

^c ^Ever, former and current ST use were compared with never ST. Use indicates timing not given and comparison is with non use.

^d ^'Id.' is the RR/OR identification number, and 'Cases' is the number of cases in ST users as defined. NA = not available.

^e ^Abbreviations used: alc = alcohol, asp = aspirin, bir = birth cohort, bmi = body mass index, edu = education, exer = exercise, occ = occupation, pov = poverty, res = area of residence, smok = smoking. NA = not available.

^f ^The population included < 0.5% females.

^g ^RR/OR and/or 95% CI estimated from data provided in the source.

^h ^Including melanoma *in situ*

CI = confidence interval; ST = smokeless tobacco; OR = odds ratio; RR = relative risk.

### Overall cancer risk

**Figure 13:**
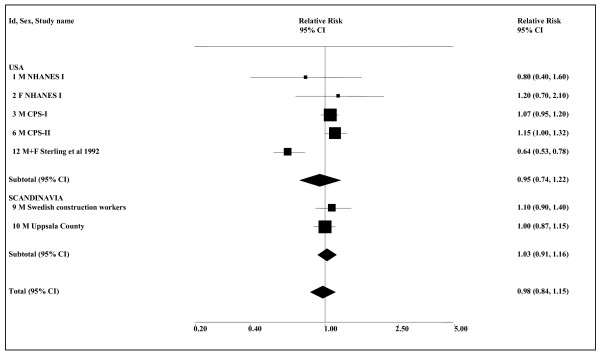
**Smokeless tobacco and overall cancer by region (smoking-adjusted data)**. The seven individual smoking-adjusted relative risk (RR) and 95% confidence interval (CI) estimates, separated by region, are shown numerically and also graphically on a logarithmic scale. They are sorted in order of year of publication within study type (cohort, case-control). In the graphical representation individual RR estimates are indicated by a solid square, with the area of the square proportional to the weight (inverse-variance) of the estimate. Also shown are the combined estimates, for the subgroups and overall, derived by random-effects meta-analysis. These are represented by a diamond of standard height, with the width indicating the 95% CI. See Table 26 for further details relating to the estimates, and Table 27 for fuller details of the meta-analyses.

As shown in Table 26, ST use has been related to overall cancer risk in five cohort studies and one case-control study. Two of the 12 estimates shown are smoking-adjusted estimates for smokers and non-smokers combined, one (estimate 10) showing no association at all (RR = 1.00) and the other (estimate 12, based on the case-control study [89]) a reduced OR of 0.64 (95% CI 0.53–0.78). The remaining 10 estimates, all from cohort studies, and all adjusted for age and various other potential confounders, are for never smokers. As shown in Table 27 and Figure 13, the combined estimate for all the smoking-adjusted data is not elevated (0.98, 0.84–1.15, *n *= 7). However, the combined estimate for never smokers, which excludes the low estimate from the case-control study, is a significant 1.10 (1.02–1.19, *n *= 6). The estimate for never smokers is similar for the US data (1.10, 1.01–1.20, *n *= 4) and the Scandinavian snuff data (1.10, 0.94–1.29, *n *= 2). The data are consistent with any excess risk of cancer in ST users being small.

**Table 26:** Overall cancer; individual effect (relative risk/odds ratio) estimates

	ST use				RR/OR			
				
Source^a^	Type^b^	Exposure^c^	Smoking	Sex	Id.	Cases^d^	Estimate (95%CI)^d^	Adjustment factors^e^
**Cohort studies**								
								
NHANES I: Accortt *et al*. 2005 [22]	ST	Ever	Never	M	1	38	0.80 (0.40–1.60)	age, pov, race
				F	2	26	1.20 (0.70–2.10)	age, pov, race
CPS-I: Henley *et al*. 2005 [23]	ST	Current	Never	M	3	357	1.07 (0.95–1.20)	age, alc, asp, bmi, diet, edu, exer, occ, race
CPS-II: Henley *et al*. 2005 [23]	ST	Current	Never	M	4	162	1.19 (1.02–1.40)	age, alc, asp, bmi, diet, edu, exer, occ, race
	ST	Former			5	57	1.04 (0.80–1.36)	
	ST	Ever			6	219	1.15 (1.00–1.32)^f^	
	Chew only	Current			7	113	1.23 (1.02–1.49)	
	Snuff only	Current			8	14	0.93 (0.55–1.57)	
Swedish construction workers: Bolinder *et al*. 1994 [28]	Snuff	Current	Never	M	9	96	1.10 (0.90–1.40)	age, res
Uppsala County: Roosaar *et al*. 2008 [35]	Snuff	Ever	Any	M	10	237	1.00 (0.87–1.15)	age, alc, res, smok, time
			Never		11	138	1.10 (0.90–1.40)	age, alc, res, time
								
**Case-control studies**								
Sterling *et al*. 1992 [89]	ST	Ever	Any	M+F	12	2,498^g^	0.64 (0.53–0.78)^f^	age, alc, occ, race, sex, smok

^a ^Fuller details of the studies are given in Tables 1 and 2.

^b ^ST implies smokeless tobacco unspecified, or combined snuff use or chewing.

^c ^Ever, former and current ST use were compared with never ST. Use indicates timing not given and comparison is with non use.

^d ^'Id.' is the RR/OR identification number used in Table 27, and 'Cases' is the number of cases in ST users as defined.

^e ^Abbreviations used: alc = alcohol, asp = aspirin, bmi = body mass index, edu = education, exer = exercise, occ = occupation, pov = poverty, res = area of residence, smok = smoking.

^f ^RR/OR and/or 95% CI estimated from data provided in the source.

^g ^Number of cases estimated from data provided in the source.

CI = confidence interval; ST = smokeless tobacco; OR = odds ratio; RR = relative risk.

**Table 27:** Overall cancer; meta-analysis results

				Heterogeneity		
				
Type of ST (region)^a^	Adjustments/restrictions^b^	Number of estimates (RR/OR ids)^c^	Random-effects RR/OR (95% CI)	χ^2^	I^2^	*P*(χ^2^)
Any	Overall data	7 (1, 2, 3, 6, 9, 10, 12)	0.98 (0.84–1.15)	27.1	77.9	< 0.001
	Smoking-adjusted	7 (1, 2, 3, 6, 9, 10, 12)	0.98 (0.84–1.15)	27.1	77.9	< 0.001
	Never smokers	6 (1, 2, 3, 6, 9, 11)	1.10 (1.02–1.19)	1.5	0.0	0.911
						
Any (USA)	Overall data	5 (1, 2, 3, 6, 12)	0.95 (0.74–1.22)	26.5	84.9	< 0.001
	Smoking-adjusted	5 (1, 2, 3, 6, 12)	0.95 (0.74–1.22)	26.5	84.9	< 0.001
	Never smokers	4 (1, 2, 3, 6)	1.10 (1.01–1.20)	1.5	0.0	0.679
						
Snuff (Scandinavia)	Overall data	2 (9, 10)	1.03 (0.91–1.16)	0.5	0.0	0.475
	Smoking-adjusted	2 (9, 10)	1.03 (0.91–1.16)	0.5	0.0	0.475
	Never smokers	2 (9, 11)	1.10 (0.94–1.29)	0.0	0.0	1.000

^a ^For each study/sex, the RR/OR for ST from Table 26 was included if available, otherwise that for chewing tobacco or snuff was used.

^b ^Smoking-adjusted includes estimates for smokers and non-smokers combined, adjusted for smoking if available, and estimates for never smokers otherwise.

^c ^The actual estimates included are identified by their RR/OR identification numbers as given in Table 26.

CI = confidence interval; ST = smokeless tobacco; OR = odds ratio; RR = relative risk.

### Publication bias

There are 49 meta-analyses presented that combine five or more effect estimates. The test of publication bias [121] shows none to be significant at *P *< 0.01, and two significant at *P *< 0.05, similar to the numbers one would expect by chance. Both the significant cases (see Tables 22 and 24) arise due to a single high effect estimate, with the other estimates included in the analysis relatively close to 1.0.

### Sensitivity analyses

Table 28 shows the effect on the smoking-adjusted analyses of successively removing those RR/OR estimates with the largest Q^2 ^values. Results are only shown for those cancers where significant (*P *< 0.05) heterogeneity was evident, and removal continues until no significant heterogeneity is seen. For pancreatic, lung and bladder cancer and for non-Hodgkin's lymphoma, only relatively high estimates are removed, and the random-effects estimate decreased, though only for lung cancer was the estimate now significantly below 1.0. For digestive cancer, the effect is to increase the estimate, but the significance is unchanged. For overall cancer, the effect is also to increase the estimate, here to marginal significance, 1.07 (1.00–1.15). For oropharyngeal cancer, the original substantial heterogeneity (*P *< 0.001) is seen to be due mainly to four estimates, three high and one low. The excess decreases from a significant 1.36 (1.04–1.77) to a non-significant 1.17 (0.95–1.45) after the removal of these estimates.

**Table 28:** Sensitivity analyses for smoking-adjusted data. Effect of removing relative risk/odds ratio estimates with largest Q^2 ^values on heterogeneity and random-effects meta-analysis estimates

Cancer (number of estimates)	RR/OR estimate removed			Heterogeneity		Random-effects RR/OR (95% CI)
			
	Id.	RR/OR	Q^2^	χ^2^	*P*	
Oropharyngeal (*n *= 19)				69.5	< 0.001	1.36 (1.04–1.77)
	35	2.67 (1.83–3.90)	15.6	52.2	< 0.001	1.27 (0.99–1.64)
	13	2.05 (1.48–2.83)	12.2	37.9	0.002	1.20 (0.94–1.52)
	43	6.20 (1.90–19.80)	8.9	28.9	0.017	1.11 (0.90–1.38)
	7	0.70 (0.50–0.90)	6.2	21.0	0.101	1.17 (0.95–1.45)
						
Pancreatic (*n *= 7)				21.2	0.002	1.07 (0.71–1.60)
	5	1.67 (1.12–2.50)	6.0	13.8	0.017	0.95 (0.63–1.46)
	1	1.70 (0.90–3.10)	4.1	9.2	0.057	0.83 (0.54–1.28)
						
Overall digestive (*n *= 5)				17.3	0.002	0.86 (0.59–1.25)
	19	0.40 (0.24–0.69)	13.3	3.1	0.382	1.14 (0.99–1.33)
						
Lung (*n *= 6)				28.7	< 0.001	0.99 (0.71–1.37)
	6	1.77 (1.14–2.74)	15.5	12.7	0.013	0.83 (0.63–1.08)
	2	6.80 (1.60–28.5)	9.4	3.3	0.343	0.72 (0.65–0.80)
						
Bladder (*n *= 10)				22.3	0.008	0.95 (0.71–1.29)
	12	1.67 (1.09–2.55)	6.9	14.3	0.074	0.86 (0.65–1.13)
						
Non-Hodgkin's lymphoma (*n *= 3)						
				9.5	0.009	1.35 (0.62–2.95)
	19	4.00 (1.30–12.0)	6.9	2.2	0.137	0.92 (0.57–1.50)
						
Overall (*n *= 7)				27.1	< 0.001	0.98 (0.84–1.15)
	12	0.64 (0.53–0.78)	21.4	2.8	0.725	1.07 (1.00–1.15)

CI = confidence interval; OR = odds ratio; RR = relative risk.

Similar analyses for the overall data (not shown) were also carried out. They also did not help to demonstrate any clear effect of ST on risk. For oropharyngeal cancer, where heterogeneity is very marked indeed, this is mainly due to estimates with atypically high values (see particularly Table 3 id. numbers 1, 15, 21, 22, 34 and 35).

Table 29 compares the smoking-adjusted meta-analysis estimates reported earlier with those recalculated preferring, where there was a choice, estimates for current ST use to those for ever use or unspecified ST use. The meta-analyses for the 12 cancers considered are based on a total of 83 effect estimates. In only 19 of these (23%) did the change in order of preference affect the estimate chosen. For 10 of these the estimate for current ST use is higher than that for ever or unspecified use, for eight it is lower, and for the other the two estimates are the same. The largest change is for pancreatic cancer in the Swedish construction workers study [32], where the selected RR value increases from 0.90 (0.70–1.20) in the original analysis to 2.10 (1.20–3.60) in the sensitivity analysis. However most of the changes, in either direction, are quite minor.

**Table 29:** Further sensitivity analyses for smoking-adjusted data. Effect of preferring estimates for current smokeless tobacco use to those for ever or unspecified smokeless tobacco use

				Heterogeneity	
				
Cancer	Analysis^a^	*N *(*nc*)^b^	Random-effects RR/OR (95% CI)	χ^2^	*P*
Oropharyngeal	Table 4	19	1.36 (1.04–1.77)	69.5	< 0.001
	Sensitivity	(5)	1.42 (1.10–1.84)	51.1	< 0.001
					
Oesophageal	Table 6	7	1.13 (0.95–1.36)	4.4	0.623
	Sensitivity	(2)	1.11 (0.92–1.34)	4.1	0.665
					
Stomach	Table 8	8	1.03 (0.88–1.20)	10.3	0.173
	Sensitivity	(2)	1.01 (0.86–1.19)	10.4	0.165
					
Pancreatic	Table 10	7	1.07 (0.71–1.60)	21.5	0.001
	Sensitivity	(2)	1.22 (0.75–2.01)	23.1	< 0.001
					
Overall digestive	Table 12	5	0.86 (0.59–1.25)	17.3	0.002
	Sensitivity	(1)	0.85 (0.57–1.27)	17.3	0.002
					
Larynx	Table 14	2	1.34 (0.61–2.95)	4.0	0.044
	Sensitivity	(1)	1.45 (0.73–2.88)	2.5	0.116
					
Lung	Table 16	6	0.99 (0.71–1.37)	28.7	< 0.001
	Sensitivity	(3)	1.11 (0.73–1.69)	20.6	< 0.001
					
Prostate	Table 18	4	1.29 (1.07–1.55)	1.2	0.764
	Sensitivity	(0)			
					
Bladder	Table 20	10	0.95 (0.71–1.29)	22.3	0.008
	Sensitivity	(1)	0.94 (0.68–1.29)	23.7	0.005
					
Kidney	Table 22	5	1.09 (0.69–1.71)	6.9	0.142
	Sensitivity	(1)	1.07 (0.60–1.91)	9.6	0.048
					
Non-Hodgkin's lymphoma	Table 24	3	1.35 (0.62–2.94)	9.5	0.009
	Sensitivity	(0)			
					
Overall	Table 27	7	0.98 (0.84–1.15)	27.1	< 0.001
	Sensitivity	(1)	0.99 (0.83–1.17)	27.9	< 0.001

^a ^For each cancer the first line repeats the original results preferring ever or unspecified ST use shown in the Table indicated, while the second line presents the results of the sensitivity analysis preferring current ST use.

^b ^N is the number of estimates included in the original and sensitivity analyses; *nc *is the number of changed estimates. For each cancer, the identification numbers for the estimates (shown in the Table indicated) included in the sensitivity analysis are shown below, with those not used in the original analysis in italic.

Oropharyngeal (Table 3): 2, 3, *4*, *8*, 11, 13, 18, 26, 35, 43, 48, 51, 55, 56, 58, *61*, *70*, 74, *75*

Oesophageal (Table 5): *3*, 6, 10, 11, 19, *20*, 23

Stomach (Table 7): 1, *4*, *7*, 10, 14, 17, 19, 21

Pancreatic (Table 9): 1, *3*, *8*, 11, 16, 18, 23

Overall digestive (Table 11): 4, 5, 6, *7*, 19

Larynx (Table 13): 3, *14*

Lung (Table 15): 2, 3, *4*, *9*, *14*, 20

Prostate (Table 17): 1, 3, 5, 7

Bladder (Table 19): *1*, 4, 8, 9, 12, 17, 21, 22, 27, 31

Kidney (Table 21): *1*, 5, 13, 17, 19

Non-Hodgkin's lymphoma (Table 23): 5, 13, 19

Overall cancer (Table 26): 1, 2, 3, *4*, 9, 10, 12

CI = confidence interval; OR = odds ratio; RR = relative risk.

For 8 of the 12 cancers, the change to the meta-analysis estimate from the altered preference is very small, by ± 0.02 at most. For oropharyngeal cancer it increases by 0.06, for larynx cancer by 0.11, for lung cancer by 0.12 and for pancreatic cancer 0.15. None of these changes materially affect the significance or the interpretation. Although there is perhaps a slight indication that associations may be stronger for current use, the tendency of most studies to report results only for ever or unspecified ST use limits the extent to which this can be investigated. Changing preferences did not materially affect the heterogeneity of the estimates. The effect of similarly changing the preference on the other meta-analyses shown earlier (for example, for never smokers or by country) also did not materially affect the results obtained (data not shown).

### Meta-regression analyses

For oropharyngeal cancer, based on the 19 smoking-adjusted estimates, where the deviance (heterogeneity χ^2^) is 69.5 (*P *< 0.001), significant reductions in deviance in 'one factor at a time' analysis are seen for period by study type (*P *< 0.001, drop in deviance 46.7 on 2 d.f.), sex (*P *= 0.020, drop 26.9 on 2 d.f.) and region (*P *= 0.014, drop 21.3 on 1 d.f.). However, the tendency for estimates to be high in females and the USA was no longer significant after adjustment for period by study type, this relationship reflecting the tendency for estimates to be high in case-control studies published before 1990, low in case-control studies published after 1990, and intermediate in prospective studies (see Figure 2).

Based on the 41 overall estimates (whether smoking-adjusted or not) for oropharyngeal cancer, where the deviance is 335.6 (*P *< 0.001), the most significant factor is sex (*P *= 0.004, drop 83.4 on 2 d.f.). Though drops in deviance of 20 or more are also seen for region, period by study type and smoking status, with estimates high for females, USA, old case-control studies and data unadjusted for smoking, no other factor is significant at *P *< 0.05 after adjustment for sex. The high deviance of 335.6 is clearly due to very high Q^2 ^values for some estimates, and further analyses were run excluding these estimates (ids 1, 7, 21, 22 and 34 in Table 3). This reduces the deviance considerably, to 84.4, though it is still highly significant (*P *< 0.001). However, again sex was the most significant factor (*P *= 0.02), with no further factor significant at *P *< 0.05 after adjusting for sex.

Meta-regression analyses were not attempted for larynx, nasal or prostate cancer or for overall digestive cancer or non-Hodgkin's lymphoma because of insufficient numbers of estimates, or for oesophageal, stomach and kidney cancer because of lack of heterogeneity. For pancreatic and bladder cancer, none of the factors investigated significantly (at *P *< 0.05) explained the heterogeneity. For overall cancer, study type was significant (*P *= 0.001), but this merely reflected the low estimate for the single case-control study, evident also in the sensitivity analysis shown in Table 28. For lung cancer, a tendency was noted for never-smoking estimates to be high, significant for both the smoking-adjusted data (*P *= 0.025) and the overall data (*P *= 0.029). This difference reflected the two high estimates already noted in the sensitivity analysis.

### Summary of meta-analyses for ST use in Western populations

Table 30 brings together all the meta-analysis results for ST use in Western populations. Based on smoking-adjusted data, significant increases (*P *< 0.05) are seen for oropharyngeal cancer, though not based on studies published since 1990, and for prostate cancer, but not for any other cancer considered. For never smokers, significant increases are seen for oropharyngeal cancer (again not when based on studies published since 1990), for oesophageal cancer and also for overall cancer. Compared with the smoking-adjusted estimates, the estimates for never smokers tend to be more variable, due to smaller numbers of ST-exposed cases studied, though they consistently exceed 1.0.

**Table 30:** Summary of meta-analyses for smokeless tobacco use in Western populations

	Overall data		Smoking-adjusted data		Never smokers	
			
Cancer	*n*	RR/OR (95% CI)	*n*	RR/OR (95% CI)	*n*	RR/OR (95% CI)
Oropharyngeal (Table 4)	41	1.79 (1.36–2.36)	19	1.36 (1.04–1.77)	9	1.72 (1.01–2.94)
- (published since 1990)	18	1.28 (0.94–1.76)	14	1.00 (0.83–1.20)	7	1.24 (0.80–1.90)
Oesophageal (Table 6)	10	1.25 (1.03–1.51)	7	1.13 (0.95–1.36)	4	1.91 (1.15–3.17)
Stomach (Table 8)	9	1.03 (0.90–1.19)	8	1.03 (0.88–1.20)	4	1.27 (0.75–2.13)
Pancreatic (Table 10)	7	1.00 (0.68–1.47)	7	1.07 (0.71–1.60)	5	1.23 (0.66–2.31)
Any digestive (Table 12)	5	0.86 (0.59–1.25)	5	0.86 (0.59–1.25)	4	1.14 (0.99–1.33)
Larynx (Table 14)	5	1.43 (1.08–1.89)	2	1.34 (0.61–2.95)	0	-
Lung (Table 16)	9	0.96 (0.73–1.27)	6	0.99 (0.71–1.37)	5	1.34 (0.80–2.23)
Prostate (Table 18)	5	1.20 (1.03–1.40)	4	1.29 (1.07–1.55)	3	1.81 (0.76–4.30)
Bladder (Table 20)	14	1.00 (0.80–1.25)	10	0.95 (0.71–1.29)	6	1.10 (0.60–2.02)
Kidney (Table 22)	11	1.23 (0.86–1.76)	5	1.09 (0.69–1.71)	2	2.19 (0.63–7.70)
Non-Hodgkin's lymphoma (Table 24)	5	1.20 (0.83–1.75)	3	1.35 (0.62–2.95)	3	1.35 (0.62–2.95)
Overall cancer (Table 27)	7	0.98 (0.84–1.15)	7	0.98 (0.84–1.15)	6	1.10 (1.02–1.19)

*n *= number of estimates included in meta-analyses.

RR/OR = combined random-effects estimate based on RRs or ORs.

CI = confidence interval; OR = odds ratio; RR = relative risk.

### Summary of meta-analyses for ST use in the USA

Table 31 similarly brings together the results for ST use in the USA. With the exception of oesophageal cancer in never smokers, significant increases seen in Table 28 are again significant here, with an increase additionally seen in the smoking-adjusted estimate for larynx cancer (although based on only a single study).

**Table 31:** Summary of meta-analyses for smokeless tobacco use in the USA

	Overall data		Smoking-adjusted data		Never smokers	
			
Cancer	*n*	RR/OR (95% CI)	*n*	RR/OR (95% CI)	*n*	RR/OR (95% CI)
Oropharyngeal (Table 4)	31	2.16 (1.55–3.02)	12	1.65 (1.22–2.25)	5	3.33 (1.76–6.32)
Oesophageal (Table 6)	6	1.56 (1.11–2.19)	3	1.89 (0.84–4.25)	3	1.89 (0.84–4.25)
Stomach (Table 8)	4	1.41 (0.95–2.10)	3	1.41 (0.93–2.12)	2	1.96 (0.82–4.70)
Pancreatic (Table 10)	5	0.86 (0.47–1.57)	5	0.99 (0.51–1.91)	3	1.09 (0.44–2.67)
Any digestive (Table 12)	5	0.86 (0.59–1.25)	5	0.86 (0.59–1.25)	4	1.14 (0.99–1.33)
Larynx (Table 14)	4	1.56 (1.21–2.00)	1	2.01 (1.15–3.51)	0	--
Lung (Table 16)	6	1.22 (0.82–1.83)	4	1.38 (0.72–2.64)	3	1.79 (0.91–3.51)
Prostate (Table 18)	5	1.23 (1.03–1.40)	4	1.29 (1.07–1.55)	3	1.81 (0.76–4.30)
Bladder (Table 20)	9	1.11 (0.85–1.45)	6	1.24 (0.83–1.85)	5	1.25 (0.69–2.26)
Kidney (Table 22)	8	1.52 (0.94–2.46)	3	1.41 (0.64–3.10)	1	4.80 (1.18–19.56)
Non-Hodgkin's lymphoma (Table 24)	3	1.45 (0.81–2.59)	2	2.07 (0.70–6.13)	2	2.07 (0.70–6.13)
Overall cancer (Table 27)	5	0.95 (0.74–1.22)	5	0.95 (0.74–1.22)	4	1.10 (1.01–1.20)

*n *= number of estimates included in meta-analyses.

RR/OR = combined random-effects estimate based on RRs or ORs.

CI = confidence interval; OR = odds ratio; RR = relative risk.

### Summary of meta-analyses for snuff use in Scandinavia

As shown in Table 32, the meta-analyses of results provide overall effect estimates that, with one exception, are never significantly increased and generally are close to 1.00. The exception is for oesophageal cancer, where the marginally significant increased RR seen in relation to snuff use for never smokers (1.92, 1.00–3.68) derives solely from the Swedish Construction Workers study [34]. In that study, no increase was seen in smoking-adjusted analyses for the whole population (1.00, 0.79–1.27). Unlike the corresponding results for the USA, where meta-analysis estimates are predominantly greater than 1.0, the estimates for snuff as used in Scandinavia are as often below 1.0 as above 1.0. Generally, the results do not suggest that snuff as used in Scandinavia has any adverse effect on cancer risk.

**Table 32:** Summary of meta-analyses for snuff as used in Scandinavia

	Overall data*		Never smokers	
		
Cancer (source)	*n*	RR/OR (95% CI)	*N*	RR/OR (95% CI)
Oropharyngeal (Table 4)	7	0.97 (0.68–1.37)	4	1.01 (0.71–1.45)
Oesophageal (Table 6)	4	1.10 (0.92–1.33)	1	1.92 (1.00–3.68)
Stomach (Table 8)	5	0.98 (0.82–1.17)	2	0.90 (0.35–2.30)
Pancreatic (Table 10)	2	1.20 (0.66–2.20)	2	1.61 (0.77–3.34)
Larynx (Table 14)	1	0.90 (0.50–1.50)	0	-
Lung (Table 16)	2	0.71 (0.66–0.76)	2	0.82 (0.52–1.28)
Bladder (Table 20)	1	0.83 (0.62–1.11)	0	-
Kidney (Table 22)	1	0.72 (0.44–1.18)	0	-
Non-Hodgkin's lymphoma (Table 24)	2	1.04 (0.54–1.98)	1	0.77 (0.59–1.01)
Overall cancer (Table 27)	2	1.03 (0.91–1.16)	2	1.10 (0.94–1.29)

* all individual estimates included in these meta-analyses are smoking-adjusted or for never smokers except for one for non-Hodgkin's lymphoma.

*n *= number of estimates.

RR/OR = combined random-effects estimate based on RRs or ORs.

CI = confidence interval; OR = odds ratio; RR = relative risk.

### Dose response data

Results relating the various cancers to dose of exposure to ST are only reported in a few studies and are not presented in detail here.

For oropharyngeal cancer, eight studies were identified that related risk to extent and/or duration of exposure. In seven of these studies, which all show no overall relationship of ST with risk in Table 3[32,55,89-91,104,113], no significant dose-response relationships are seen. It was only in one study [61], that did show a clear overall relationship, that a significant (*P *< 0.001) trend in risk with increasing duration of exposure is seen, though only for cancers of the gum and buccal mucosa, and not for other mouth and pharynx cancers.

For other cancer sites relatively few studies report dose-response data. In the CPS-II study [23] no trends with duration or frequency are seen for either total or lung cancer, while in the Swedish Construction Workers study no trend is seen for cutaneous squamous cell carcinoma with years of snuff dipping [29] or for oral cancer or lung cancer with daily amount of snuff consumed [32]. A significant trend (*P *< 0.01) is reported with daily amount of snuff consumed for pancreatic cancer [32] in never smokers, but this merely reflects the overall relationship, with RRs similar in light and heavy users (1.9 for 1–9 g/day, and 2.1 for 10+ g/day relative to never users). For some of the case-control studies considered [38,44,53,55,89,96,100,104,108,110,111,114], dose-response results are available, but these generally show no significant trends. The only exceptions are a study of kidney cancer [100] which reports a significant (*P *< 0.05) trend for risk to increase with frequency of use of chewing tobacco, and a study of pancreatic cancer [111] which reports a significant (*P *= 0.04) trend for risk to increase with ounces per week (oz/wk) ST used, though with the odds ratios forming an erratic pattern (1.0 for nonusers of tobacco, 0.3 for ≤ 2.5 oz/wk ST and 3.5 for > 2.5 oz/wk ST). Generally the rather sparse dose-response data add little to the overall evidence.

### Comparison of the effects of smoking and of ST use

Table 33 summarises the results of analyses comparing the effects of smoking and of ST use, for seven smoking-related cancers [127]. Overall in US men aged 35+ a total of 142,205 deaths were seen from these cancers in 2005, with lung cancer (63.4%) by far the most common. Based on RRs from CPS-II for current and former smoking [122] and estimates of the frequency of current and former smoking [124] for US men of this age group, the total number of deaths that would have occurred if the men had the mortality rates of never smokers can be estimated as 37,468, a reduction (E) of 104,737 deaths. This reduction is proportionately largest for the cancers most strongly associated with smoking (lung and oropharynx), and least for those most weakly associated (pancreas, kidney and bladder).

**Table 33:** Comparison of effects of smoking and smokeless tobacco on smoking-related cancer^a ^in US males aged 35+

	Oropharynx	Oesophagus	Pancreas	Larynx	Lung	Bladder	Kidney	Total
Number of deaths (D_i_)^b^	5,224	10,578	16,105	2,980	90,096	9,181	8,041	142,205
								
Relative risks^c^								
Current cigarette smoking (R_ci_)	27.48	7.60	2.14	10.48	22.36	2.86	2.95	
Former cigarette smoking (R_fi_)	8.80	5.83	1.12	5.24	9.36	1.90	1.95	
								
Deaths if all the population were never smokers (D_i_*)^d^	567	2,681	12,524	679	10,901	5,445	4,671	37,468
								
Deaths eliminated if all the population were never smokers (E)^e^								104,737
								
Relative risks^f^								
- any ST use (R_si_)	1.36	1.13	1.07	1.34	1.00^g^	1.00^h^	1.09	
								
Deaths in a population of never smokers^i^								
Same % become ST users as were smokers (D_i_**)	676	2,866	12,988	801	10,901	5,445	4,894	38,570
100% of population become ST users (D_i_***)	772	3,029	13,400	910	10,901	5,445	5,091	39,548
								
Increase in deaths in a population of never smokers^j^								
Same % become ST users as were smokers (I_1_)								1,102
100% of population become ST users (I_2_)								2,081

^a ^ICD 10^th ^revision codes [127] used are oropharynx (C00–C14), oesophagus (C15), pancreas (C25), larynx (C32), lung (C33, C34), bladder (C67) and kidney (C64–C66, C68).

^b ^Numbers of deaths in 2005 from WHO [123]. Here and for other results, the entries in brackets correspond to the notation used in the methods section.

^c ^Relative risks from US Surgeon General's Report 1989 [122] Table 6 p 150, derived from CPS-II.

^d ^D_i_* is calculated as shown in the methods section, assuming 21.8% of current smokers and 31.2% of former smokers, based on the National Health Interview Survey 2005 [124].

^e ^E = Σ (D_i _- D_i_*).

^f ^Relative risks for any ST use, based on smoking-adjusted data, as given in Tables 4, 6, 8, 10, 16, 20 and 22 for the seven cancers shown.

^g ^Actually 0.99, but taken as 1.00 for the purposes of estimation.

^h ^Actually 0.95, but taken as 1.00 for the purposes of estimation.

^i ^D_i_** and D_i_*** are calculated as shown in the methods section, assuming that 53.0% or 100.0% respectively of the population use ST.

^j ^I_1 _= Σ (D_i_** - D_i_*), I_2 _= Σ (D_i_*** - D_i_*)

The smoking-adjusted relative risks for any ST use taken from Table 30 are then used to estimate the number of deaths that would have occurred if the population were never smokers, with ST use either at the same frequency as for current and former smoking combined, 53%, or at 100%. In the first situation, the number of cancer deaths rises from 37,468 to 38,570, an increase of 1,102; in the second situation, it rises to 39,548, an increase of 2,081. These numbers of cancers associated with ST use form, respectively, 1.1% and 2.0%, of E, the number associated with smoking.

## Discussion

### Estimating the effects of ST use

We have analysed data relating cancer risk to the consumption of chewing tobacco and snuff as used in Western countries. We have identified 12 cancers (or combined categories) where, as shown in Table 30, it is possible to derive a (random-effects) meta-analysis estimate based on at least five individual independent estimates.

It is notable that no strong association at all is evident and that few of the associations are significant at *P *< 0.05. Indeed, based on smoking-adjusted data, which might be argued to provide a good compromise between avoidance of bias and loss of power, only the estimates for oropharyngeal and prostate cancer are significant, with that for oropharyngeal cancer not evident in more recently published studies. However, it should be noted that while many of the estimates in Table 30 for never smokers have wide confidence limits, and only those for oropharyngeal and oesophageal cancer and for overall cancer are significant, all the estimates are in fact greater than 1.00. Although publication bias may be relevant, and more data are clearly needed, the consistency of these findings suggests that ST may increase the risk of cancer, though any effect is likely to be quite weak. The results in Table 32 suggest, however, that whether smoking-adjusted data or data for never smokers are considered, there is little or no evidence of an effect of snuff as used in Scandinavia.

There are a number of difficulties in interpreting the results of these meta-analyses. The studies are of varying design, size and quality. Many of the individual study reports have limitations and present less information than is ideal for a meta-analysis. Shortcomings include small numbers of cases, and in particular of cases exposed to ST, lack of histological confirmation, lack of division by cancer site, as well as an unclear description of inclusion and exclusion criteria, details of case and control selection, and methods of exposure assessment. Furthermore, details such as the type of ST used, and duration and frequency of use, are often not considered. The products used vary by country and over time, and increased risks seen in older studies for some cancers may not reflect the risks of more modern products, with reduced nitrosamine levels [128]. For most cancers, the number of effect estimates available is really too limited to allow a very detailed examination of variation in risk by such factors as type of product used, current or former use, country and sex. Though meta-regressions have been attempted for a number of cancers, they have not added materially to the interpretation, partly because of the limited amount of data for some cancers, and partly because of the number of apparently outlying estimates, notably for oropharyngeal cancer.

A major problem is that many of the studies fail to adjust for smoking and other important potential confounding variables. Although recent major reviews [7,8] consider that all the cancers considered in Table 30, with the exception of prostate cancer and non-Hodgkin's lymphoma, are caused by smoking, it is evident that a number of the studies do not provide estimates that are either for never smokers or for smokers and non-smokers combined with adjustment for smoking. Even where adjustment for smoking is carried out, this is often by a relatively simple approach, with no account taken of number of cigarettes smoked or duration of smoking. Smokers who also use ST may smoke fewer cigarettes a day than smokers who do not. Failure to adjust for smoking is particularly common for studies of oropharyngeal cancer, with many of the older studies not taking smoking into account at all when considering ST. The potential importance of this is illustrated by the overall estimate for oropharyngeal cancer being substantially reduced, from 1.79 to 1.36, when attention is restricted to smoking-adjusted data.

Adjustment for other risk factors is also important, as shown by the case of oropharyngeal cancer where the smoking-adjusted estimate of 1.36 (1.04–1.77, *n *= 19) can be compared with the estimate adjusted for smoking and alcohol of 1.07 (0.84–1.37, *n *= 10). Restricting attention to estimates adjusted for both factors also eliminated the highly significant (*P *< 0.001) heterogeneity seen in the smoking-adjusted data. Alcohol is also an important factor in the aetiology of oesophageal, larynx and liver cancer [8], but the number of ST effect estimates adjusted both for smoking and alcohol for these three cancers is very low indeed, respectively 2, 1 and 0. Other factors considered rarely, or not at all, include, for example, *Helicobacter pylori *infection for stomach cancer and diet for digestive cancer.

Another difficulty in interpreting the overall results is the variability of the findings. Heterogeneity significant at least at *P *< 0.05 is evident in the smoking-adjusted estimates for cancers of the oropharynx (though not in the more recent data), pancreas, larynx, lung and bladder, as well as for overall cancer and overall digestive cancer. As noted above, the evidence is too limited for most of the cancers to allow a proper investigation of the sources of this heterogeneity.

Based on the data analysed, there is little or no evidence of publication bias. However, it should be noted that the number of studies reporting results in a form that cannot be included in the meta-analyses is fairly high, representing up to about 30% for some cancers (see Tables 5, 7, 9, 13 and 17).

We are aware that the smoking-adjusted meta-analysis estimates we report for oropharyngeal cancer (1.36, 95% CI 1.04–1.77)), oesophageal cancer (1.13, 0.95–1.36), pancreatic cancer (1.07, 0.71–1.60) and lung cancer (0.99, 0.71–1.37) show much less evidence of a relationship with ST than do corresponding estimates recently reported in a review by Boffetta *et al*. [6] (oropharynx: 1.8, 1.1–2.9; oesophagus: 1.6, 1.1–2.3; pancreas: 1.6, 1.1–2.2; lung 1.2, 0.7–1.9). Reasons for this, based on a detailed analysis of this review, will be presented in a separate publication in BMC Cancer.

### Comparison of the effects of smoking and ST use

In 2005 in US men aged 35 or over, there were a total of 142,205 deaths from seven cancers considered to be caused by smoking. Based on relative risks from CPS-II for current and former smoking [122] and estimates of the frequency of current and former smoking [124] for US men of this age group, we estimate that, had the population at risk the mortality rates of never smokers, the numbers would have reduced by 104,737, with the reduction in lung cancer deaths, 79,195, a major contributor. Any increase in risk resulting from the introduction of ST to a population of never smokers would be very much less than this. Even assuming that the smoking-adjusted meta-analysis estimates for the seven cancers all reflect a true effect of ST, the increase in deaths among a never-smoker population would be by 1,102 if 53% of the population used ST (the same proportion as had ever smoked) or by 2,081 if the whole population did. These increases represent, respectively, only 1.1% and 2.0% of the 104,737 deaths attributed to cigarette smoking.

There are a number of objections that can be made in respect of this comparison. These include the following:

1. The RRs for current and former smoking are based on CPS-II, conducted in the 1980s, and may not reflect those appropriate for 2005, given *inter alia *changes in cigarettes that have occurred since then. However, CPS-II is widely used as a source of data for calculating deaths attributed to smoking (for example, [8,129]).

2. The RR estimates used for ST use are not specifically for the USA, or for males. However, 62 of the 89 studies considered in this review were conducted in the USA, and 41 of the 58 estimates used in the smoking-adjusted meta-analyses for the seven cancers are for males (with 12 for sexes combined and five for females).

3. The RR estimates used for ST are for any ST use, and do not separate current and former use, due to most studies not providing such data.

4. The calculations are limited to those seven cancers which the US Surgeon General, in his 1989 report [122] considered to be caused by smoking and for which RRs were provided for CPS-II. A more recent report [8] includes stomach cancer and leukaemia as caused by smoking. For stomach cancer, the meta-analyses in Table 6 showed virtually no association with ST use (1.03, 0.88–1.20, *n *= 8), while the more limited data for leukaemia also showed no clear evidence of a relationship.

5. It is theoretically possible that ST use might increase the risk of some cancers not increased by smoking. Here one should note the significant association for prostate cancer (1.29, 1.07–1.55).

6. The calculations do not take into account the fact that a proportion of US males aged 35+ already use ST. Given the relatively weak association between cancer and ST use, any attempt to do this would have had relatively little effect.

7. The calculations also do not take pipe and cigar smoking into account.

8. The approach used is somewhat simplistic, and a more realistic (but more complex) calculation might be to compare predicted cancer deaths over a long-term period in a population continuing to smoke as at present, with the predicted number in a population switching from cigarettes to ST.

Despite all these points, it is clear that any effect of ST on risk of cancer, if it exists at all, is quantitatively very much smaller than the known effects of smoking. This is in any case apparent from a simple comparison of the RRs for cigarette smoking and for ST use.

## Conclusion

The available data relating to ST use have a number of weaknesses, including inadequate control for smoking in many, and limited data for never smokers. Nevertheless, it is possible to conduct meta-analyses based on smoking-adjusted estimates for a relatively wide range of cancers. These show no indication of an increased risk of cancer for snuff, as used in Scandinavia. The overall data for oropharyngeal cancer shows a significant increase in risk associated with ST use, but this is not evident for estimates adjusted for smoking and alcohol, or for studies published since 1990. Any effect of ST may relate mainly to products used in the past in the USA. A weak but significant association with prostate cancer, based on limited data from US studies, requires more confirmatory evidence. Reports of significant associations with pancreatic and oesophageal cancer in an earlier review [6] are not confirmed, and reasons for this will be discussed in a later publication. Risk from ST products as used in North America and Europe is clearly very much less than that from smoking, and is not evident at all in Scandinavia.

## Abbreviations

CI: 95% confidence interval; CPS-I: American Cancer Society Cancer Prevention Study I; CPS-II: American Cancer Society Cancer Prevention Study II; d.f.: degrees of freedom; NHANES I: First National Health and Nutrition Examination Survey; OR: odds ratio; RR: relative risk; ST: smokeless tobacco.

## Competing interests

PNL, founder of PN Lee Statistics and Computing Ltd., is an independent consultant in statistics and an advisor in the fields of epidemiology and toxicology to a number of tobacco, pharmaceutical and chemical companies. JH works for PN Lee Statistics and Computing Ltd.

## Authors' contributions

PNL previously contributed to reviews of some of the data considered here [4,5,10]. He planned the study and carried out the literature search. PNL and JH jointly extracted the estimates and conducted the meta-analyses. The text and tables were drafted by PNL and checked by JH. Both authors read and approved the final manuscript.

## Pre-publication history

The pre-publication history for this paper can be accessed here:

http://www.biomedcentral.com/1741-7015/7/36/prepub
